# Innovative Functional Biomaterials as Therapeutic Wound Dressings for Chronic Diabetic Foot Ulcers

**DOI:** 10.3390/ijms24129900

**Published:** 2023-06-08

**Authors:** Jessica Da Silva, Ermelindo C. Leal, Eugénia Carvalho, Eduardo A. Silva

**Affiliations:** 1 CNC—Center for Neuroscience and Cell Biology, CIBB—Center for Innovative Biomedicine and Biotechnology, University of Coimbra, Rua Larga, 3004-504 Coimbra, Portugal; jessicasilva@cnc.uc.pt (J.D.S.); ecleal@cnc.uc.pt (E.C.L.); 2 PDBEB—Ph.D. Programme in Experimental Biology and Biomedicine, University of Coimbra, 3004-504 Coimbra, Portugal; 3 Institute of Interdisciplinary Research, University of Coimbra, Casa Costa Alemão, Rua Dom Francisco de Lemos, 3030-789 Coimbra, Portugal; 4 Department of Biomedical Engineering, Genome and Biomedical Sciences Facilities, UC Davis, 451 Health Sciences Dr., Davis, CA 95616, USA; eduardo.silva@uis.no; 5 Department of Chemistry, Bioscience, and Environmental Engineering, University of Stavanger, Kristine Bonnevies vei 22, 4021 Stavanger, Norway

**Keywords:** biomaterials, chronic wounds, clinical translation, diabetic foot ulcers, natural and synthetic materials, wound dressings, wound healing

## Abstract

The imbalance of local and systemic factors in individuals with diabetes mellitus (DM) delays, or even interrupts, the highly complex and dynamic process of wound healing, leading to diabetic foot ulceration (DFU) in 15 to 25% of cases. DFU is the leading cause of non-traumatic amputations worldwide, posing a huge threat to the well-being of individuals with DM and the healthcare system. Moreover, despite all the latest efforts, the efficient management of DFUs still remains a clinical challenge, with limited success rates in treating severe infections. Biomaterial-based wound dressings have emerged as a therapeutic strategy with rising potential to handle the tricky macro and micro wound environments of individuals with DM. Indeed, biomaterials have long been related to unique versatility, biocompatibility, biodegradability, hydrophilicity, and wound healing properties, features that make them ideal candidates for therapeutic applications. Furthermore, biomaterials may be used as a local depot of biomolecules with anti-inflammatory, pro-angiogenic, and antimicrobial properties, further promoting adequate wound healing. Accordingly, this review aims to unravel the multiple functional properties of biomaterials as promising wound dressings for chronic wound healing, and to examine how these are currently being evaluated in research and clinical settings as cutting-edge wound dressings for DFU management.

## 1. Introduction

Diabetes mellitus (DM) represents a rapidly growing global health challenge with significant socioeconomic impacts, and its incidence is predicted to keep rising over the coming decades [1,2,3]. According to the International Diabetes Federation, DM prevalence keeps increasing, posing dramatic challenges to patients, families and societies [1,2]. In 2021, DM affected one in every ten adults (20–79 years), for a total of 537 million people worldwide [1]. Projections further indicate that this number could rise to over 783 million by 2045, a 20% increase [1]. Moreover, DM is responsible for one death every 5 s and for a 316% increase in the health expenditure over the last 15 years (USD 966 billion) [1].

**Figure 1:**
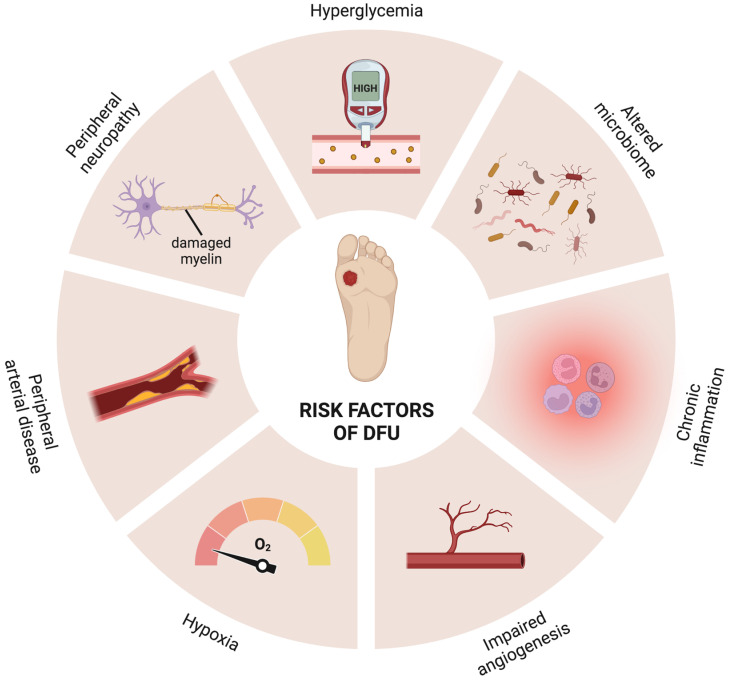
Risk factors associated with the development of diabetic foot ulcers (DFUs) (produced using BioRender). The chronic low-grade inflammation and prolonged hyperglycemia in individuals with diabetes can foster myelin damage over time, prompting peripheral neuropathy. This results in oxidative stress and impairment of the sensitive, motor, and autonomous nerves. In turn, peripheral arterial disease (PAD) leads to insufficient blood supply and subsequent hypoxia, as well as hypercoagulability and serious limb ischemia, weakening the lower-extremity zones and making them prone to secondary infections. On top of that, angiogenesis is also reduced in individuals with diabetes. Foot ulceration is hence expectable.

Individuals with DM face many risk factors that compromise their overall health and well-being, leading to a range of comorbidities, including cardiovascular diseases, diabetic retinopathy and kidney disease, nerve and/or vascular damage, and diabetic foot complications [1,4]. In particular, the risk factors involved in diabetic foot development include persistent hyperglycemia, chronic inflammation, hypoxia, peripheral neuropathy, peripheral arterial disease (PAD), impaired angiogenesis, and difficulty in fighting infections [5,6,7,8,9], as depicted in Figure 1.

Affecting 15 to 25% of individuals with DM during their lifetime, diabetic foot ulcers (DFUs), as complex lesions of the lower extremities, are one of the most significant and devastating complication of diabetes [3,5,8,10]. These ulcers might not heal over time and can develop polymicrobial infections, leading to prolonged hospitalizations and even amputations in 85% of cases [2,5,9,11,12]. Current DFU treatments focus on multidisciplinary approaches, including key aspects of diabetic wound care such as glycemic control, adequate arterial supply, the debridement of necrotic tissue, pressure offloading, and the treatment of any infection [3,4,9,11,12,13,14,15,16]. However, more effective treatment options are lacking to achieve actual efficient management of this condition, and consequently to reach superior treatment success rates [17,18].

Biomaterials are natural or synthetic materials designed to interact with biological systems [19,20], which exhibit great potential for diabetic wound healing due to their unique inherent characteristics [21,22,23,24]. On the one hand, biomaterials have been extensively explored due their ability to absorb wound exudates while providing a moist and warm environment, that are beneficial for tissue regeneration [21,22,23,24]. On the other hand, many biomaterials have also been revealed to have antimicrobial properties that help prevent infections and consequently promote proper wound healing [21,22,23]. Furthermore, biomaterials can also be used as local depots of bioactive agents that can be delivered in a controlled and sustained manner over time, reducing the risks of dose-related toxicity while promoting wound closure [21,22,23,24,25]. Owing to their versatility, biocompatibility, biodegradability, and hydrophilicity, biomaterials have arisen as encouraging candidates for the successful treatment of DFUs [22,23,24].

This review aims to unravel the multiple functional properties of biomaterials as promising wound dressings for chronic wound healing. Additionally, it will examine cutting-edge wound dressings that are undergoing clinical trials for DFU management.

## 2. Diabetic Foot Ulcer: A Multifactorial Emerging Issue

DFUs are deep tissue lesions on the lower extremities, mainly associated with sustained hyperglycemia, peripheral neuropathy, and PAD [5,6,7,8,9]. Globally, a lower limb is amputated every 20 to 30 s, with DFU being responsible for 85 to 95% of cases [8,26,27,28]. Furthermore, individuals with DFUs typically display an increased risk of mortality, more than the double risk of those with DM without a DFU [3,8,29,30]. A 5-year lower survival rate was also revealed for individuals with a DFU when compared to those with DM without DFUs [8,30,31,32]. This lower survival rate is in addition to the reduction of 6 years in life expectancy that is observed for the condition of DM itself [30,31,32].

### 2.1. Pathophysiology of Diabetic Foot Ulcers

Peripheral neuropathy is the primary predisposing factor in DFU development, due to long-term hyperglycemia, which results in oxidative stress and damages the sensitive, motor, and autonomous nerves [10,28]. Sensory defects manifest as a loss of sensitivity to injury and stimulation in the lower limbs due to small-fiber nerve dysfunction, thereby promoting constant unconscious trauma and subsequent ulceration [10]. These sensory defects may include sensory dullness, numbness, and abnormal pain, among others [10,26]. By generating intrinsic muscle weakness and atrophy, consequently leading to biomechanical anatomical changes in the feet such as hammer toe, Charcot’s ankle, pes-planus, and pes-cavus, motor neuropathy triggers high-pressure zones in the feet [3,8,10,26,28]. This increased shear stress and friction force further promote foot ulceration [10]. Peripheral sympathetic nerves may also be damaged, causing thermoregulatory dysfunction that involves altered sweating, dry skin, cracking, and calluses, subsequently facilitating ulceration [10,26].

PAD is another key factor in ulcer development, characterized as a chronic arterial occlusive disease of the lower extremities [3,10,28]. Specifically, approximately 80% of individuals with DFUs initially experience PAD, resulting in an insufficient blood supply, hypercoagulability, and serious limb ischemia [3,10,28,33]. Tissue ulceration is therefore anticipated due to long-term ischemia and hypoxia, which weaken lower-extremity regions and render them susceptible to secondary infection [3,10,28].

Significantly, 50 to 60% of DFUs become infected, predominantly with bacterial colonies of *S. aureus*, *C. striatum*, and *P. aeruginosa*, and fungal colonies of *C. albicans* [5,34,35,36,37,38]. Moreover, between 20 and 25% exhibit deep infections with some anaerobic bacteria, such as *Bacteroides* spp., *Prevotella* spp., and *Clostridium* spp., which can spread to the bone, further exacerbating the risk of mortality and the socioeconomic burden [8,28,39].

### 2.2. Impaired Healing in Diabetic Foot Ulcers

DFUs may arise from several risk factors that collectively impair the wound healing process of these individuals. Wound healing is a process with four overlapping phases, involving a complex and dynamic sequence of cellular and biochemical events to restore skin integrity and functionality after trauma [5,40,41,42]. The first phase—hemostasis—begins immediately after skin injury with the constriction of the damaged blood vessels and activation of platelets [5,40,43]. This promotes platelet aggregation and the subsequent formation of a fibrin clot, covering the injured endothelium and consequently stopping the bleeding [5,40,43]. The second phase—inflammation—starts with the recruitment of neutrophils to the wound site, as a first line of defense against pathogens [5,40,41,43]. After a peak population between 24 and 48 h post skin injury, the number of neutrophils greatly reduces and pro-inflammatory M1 phenotype macrophages arrive successively at the wound site to continue clearing microbial pathogens and debris [5,40,41]. In detail, M1 macrophages attract different types of adaptive immune system cells to the wound site by the secretion of cytokines and chemokines, either to continue clearing cellular debris or to fight infection [5,40,41,42]. These macrophages then switch from the pro-inflammatory M1 to anti-inflammatory M2 phenotype as inflammation resolves to further foster tissue regeneration, producing anti-inflammatory cytokines and growth factors. The third phase—proliferation—occurs with the formation of granulation tissue to fill the wound, the contraction of the wound borders, wound coverage with epithelial cells (i.e., re-epithelialization), and neovascularization [5,40,41,42,43]. The fourth phase—remodeling—involves collagen fiber reorganization, tissue remodeling and maturation, and an overall increase in tensile strength [5,40,41,43].

**Figure 2:**
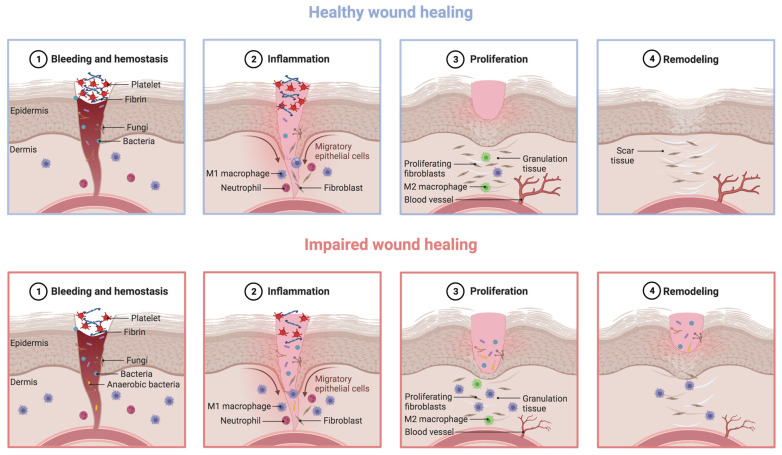
Healthy versus impaired phases of the healing process: the case of diabetic foot ulcers (DFUs) (produced using BioRender). Under DFU conditions, an unbalanced accumulation of immune cells and an increase in the M1/M2 macrophage ratio, reactive oxygen species (ROS), and pro-inflammatory cytokines occur. In addition, re-epithelialization and angiogenesis are scarce, altogether ending in chronic non-healing wounds that remain in a state of low-grade inflammation.

Nonetheless, the impairment of local and systemic factors in individuals with DFUs leads to a poorly orchestrated cascade of the four phases, thus delaying or even interrupting the healing process, as per Figure 2. The concurrent presence of DM and DFU stimulates an unbalanced accumulation of immune cells, as well as an increase in the M1/M2 macrophage ratio, reactive oxygen species (ROS), and pro-inflammatory cytokines, together ending in chronic non-healing wounds that remain in a state of low-grade inflammation [5,16,26,42]. In detail, Erem et al. showed that patients with DM exhibited hypercoagulability and decreased fibrinolysis during the hemostasis phase, compared to healthy individuals [44]. Patients with DM have also been associated with an imbalance in cytokine release by neutrophils during the inflammatory phase, thus favoring wound infection [26,45]. Furthermore, fibroblast and keratinocyte migration, as well as their proliferative capacity, is compromised in patients with DM due to hyperglycemia, leading to poor re-epithelialization of the wound [46,47]. On top of this compromised cell migration, angiogenesis is also reduced in patients with DM, resulting in decreased blood supply to the wound site [48]. During the remodeling phase, patients with DM have also shown altered fibroblast function, contributing to flawed closure of the wound [49]. This may probably be explained by an inefficient response to transforming growth factor beta (TGF-β) from fibroblasts, as well as an aberrant production of the extracellular matrix [26,49].

### 2.3. Management of Diabetic Foot Ulcer

Current therapeutic approaches for managing DFUs involve multidisciplinary strategies that address key aspects of diabetic wound care, including glycemic control, adequate arterial supply, the debridement of necrotic tissue, pressure offloading, and the treatment of any infection with appropriate broad-spectrum antibiotics [3,4,9,11,12,13,14,15,16]. For example, it was recently reported that the MADADORE acronym corresponds to the recommended DFU management principles: Metabolic control, Assessment of foot, Debridement, Antibiotics, Dressing, Offloading pressure, Referral to multidisciplinary teams, and Education [50]. Metabolic control involves the management of associated medical conditions such as hyperglycemia and hyperlipidemia, through adequate medication and dietary counseling [51]. Assessment of foot concerns the correct evaluation of the associated risk factors and classification of the ulcer according to the perfusion, extent, depth, infection, and sensation (PEDIS) scale [52]. Debridement involves the surgical removal of any necrotic or unhealthy tissue, while treating any infection with appropriate broad-spectrum antibiotics. Furthermore, dressings are needed to foster wound exudate absorption and create a protected environment propitious for tissue regeneration. In turn, offloading through minimally invasive surgery such as the minimally invasive metatarsal osteotomies is essential to reduce plantar pressure, while supporting minimal tissue damage, immediate post-operative weight bearing, and a lower risk of potential infections, consequently preventing recurrent ulceration [53,54]. Last but not least, referral to multidisciplinary teams means the indication of appropriate adjuvant therapies for the optimal management of DFUs, such as stress-reducing approaches [55,56], while education is fundamental to improve DFU health literacy for the prevention of future ulcers.

However, despite these efforts, the efficient management of DFUs remain a clinical challenge, with limited success rates in treating severe infections [17,18]. Indeed, current DFU treatments still exhibit a huge recurrence rate of 40% within one year, 60% within three years, and 65% within five years [8,13,28,57], due to persistent risk factors even after the former ulcer has healed [58]. Consequently, there is a crucial need for novel strategies that can successfully address the multifactorial etiology of DFUs.

## 3. Biomaterials as a Promising Therapeutic Platform for Wound Dressings

**Figure 3:**
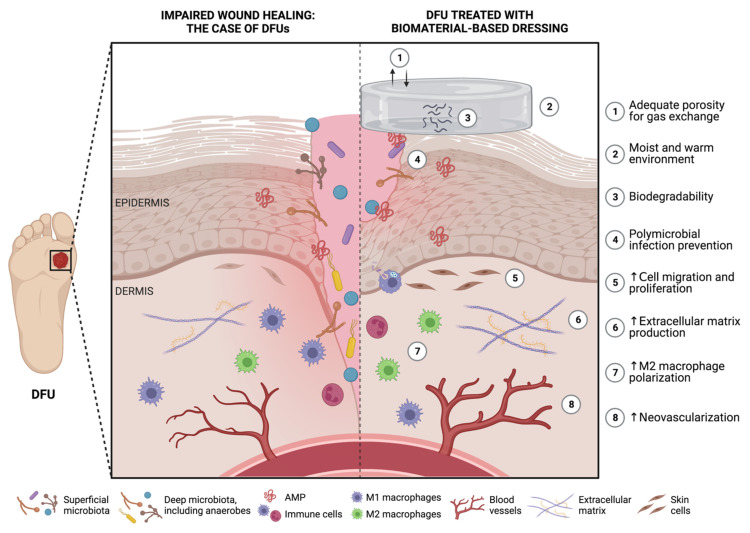
Potential features of functional biomaterials as ideal wound dressings for diabetic foot ulcer (DFU) healing (produced using BioRender). ↑ represents an increase.

A therapeutic strategy with rising potential to handle the challenging macro and micro wound environment of individuals with DM involves the use of biomaterials as wound dressings. Biomaterials have long been related to unique versatility, biocompatibility, biodegradability, and hydrophilicity, characteristics that make them ideal candidates for therapeutic applications [22,23,24]. Furthermore, biomaterials have also been explored for their innate properties for wound healing [21,22,23,24]. An ideal wound dressing for the management of DFUs should present several key features. Firstly, it must demonstrate excellent biocompatibility and biodegradability to ensure tissue healing. Secondly, it should create a moist and warm environment conducive to tissue regeneration. Thirdly, the dressing should prevent polymicrobial infections to ensure proper wound healing. Finally, it should exhibit adequate porosity that enables gas exchange, and stimulate cell migration, proliferation, and neovascularization, as depicted in Figure 3 [21,22].

### 3.1. Natural Biomaterials

Wound dressings may be produced either from naturally occurring materials or synthetic materials [24,59,60]. Common natural materials include alginate, cellulose, chitosan, collagen, dextran, fibrin, gelatin, hyaluronic acid (HA), and pectin. These materials have been widely used as biomaterials due to their excellent biocompatibility, biodegradability, and low antigenicity, making them less likely to trigger inflammatory responses [24,59,61]. Moreover, these natural biomaterials can mimic the native tissue structure and function, allowing greater cell attachment and infiltration, and further supporting tissue regeneration [24]. However, there are some limitations to their clinical use, such as their poor mechanical properties, usually requiring cross-linking with synthetic polymers that exhibit good mechanical properties, or chemical modifications to improve their intrinsic characteristics, as described in the following subsections [24,59,61]. Natural materials that have been recently evaluated in research settings as promising wound dressings and their respective formulation and main properties are summarized in Table 1.

#### 3.1.1. Alginate

Alginate is a natural biomaterial that has attracted the greatest attention in biomedical applications, along with chitosan [80,81,82]. It is composed of two linear co-polymers: (1,4)-linked β-D-mannuronic acid (M) and α-L-guluronic acid (G). It is a structural protein that is typically extracted from brown seaweeds, but that is also produced by few bacterial species including *Pseudomonas aeruginosa* [80,81,83,84,85]. The M/G ratio and the length of the chain influence its properties as a wound dressing [80,81]. Indeed, higher G content results in strong and firm gels, whereas higher M content leads to weak and soft gels [81,85,86]. Alginate’s anionic nature provides excellent biocompatibility, hydrophilicity, and biodegradability, as well as great swelling capacity, ease of gelation, pH sensitivity, and non-toxicity [81,84,85,87]. Specifically related to wound healing, it may enhance ion exchange with wound exudate and blood, while forming a protective barrier that maintains an optimal moisture content and temperature favorable for wound healing [80,82]. However, alginate also displays some drawbacks to its clinical use, such as low structural stability under physiological conditions, and no antimicrobial activity [83]. These limitations can be counteracted through chemical modifications, cross-linking with other materials, or loading with bioactive agents [83,88]. For example, Chen et al. designed an oxygen-producing patch filled with sodium alginate gel beads containing active *S. elongatus*, a unicellular cyanobacterium that has rapid autotrophic growth, to produce dissolved oxygen [62]. In detail, oxygen needs to be dissolved, i.e., to leave the gaseous phase and enter the liquid phase, to become biologically available and be diffused into a cell [89]. Accordingly, this patch delivered oxygen by penetrating the skin much more efficiently than common topical gaseous oxygen therapy, thus improving chronic wound healing and skin graft survival in diabetic mice [62]. Other authors applied the ionic cross-linking method with calcium ions to produce sodium alginate hydrogels loaded with deferoxamine (DFO) and copper nanoparticles (Cu-NPs) [63]. The simultaneous release of DFO and Cu-NPs from sodium alginate hydrogels synergistically stimulated hypoxia-inducible factor 1 alpha (HIF-1α) levels and vascular endothelial growth factor (VEGF), enhancing angiogenesis, reducing chronic inflammation, and accelerating wound healing in a diabetic mouse model [63]. Another study combined the cross-linking of sodium alginate with carboxymethyl chitosan (CMCS) and loading with a small molecular probe (Ir-fliq) based on an iridium complex to form a gel for optical imaging and photodynamic antimicrobial chemotherapy (PACT) [64]. Under light irradiation, the formed hydrogels promoted the healing of *S. aureus*-infected chronic wounds in diabetic mice, by inhibiting bacteria growth through PACT [64].

#### 3.1.2. Cellulose

Cellulose is a natural polysaccharide that is widely distributed and abundant in nature, namely in plants, fungi, algae, and bacteria [59,60]. This material has been used for wound healing applications as a biomaterial due to its good biocompatibility and hydrophilicity, which are critical features to ensure wound exudate absorption and a favorable moist environment conducive to wound healing [59,60]. Lot-to-lot variability is typically present due to the diversity of extraction sources. For instance, bacterial cellulose displays better biocompatibility, porosity, air permeability, moisture absorption, water retention, mechanical properties, and flexibility compared to cellulose of plant origin [60]. Despite these favorable characteristics, poor cell adhesion, a lack of antibacterial activity, and low water stability have been linked to cellulose, which limit its clinical translation [60]. Nonetheless, chemical modifications or blending with other materials can be used to bypass some limitations [60,90,91,92]. Carboxymethyl cellulose (CMC) is an alternative that has gained particular attention, due to its low cost and high abundance [60]. Moreover, Azarniya et al. developed composite scaffolds consisting of nanofibrous mats made of bacterial cellulose and polyethylene oxide (PEO)-modified keratin, and tragacanth gum (TG)-conjugated hydrogels [65]. The modified nanofibers with TG-conjugated hydrogels showed superior overall mechanical properties such as hydrophilicity, elasticity, tensile strength, and ductility [65]. In addition, good biocompatibility with L929 fibroblasts was observed, as well as enhanced cell adhesion and proliferation, thus underlining the potential of this hybrid composite scaffold as an attractive wound dressing [65]. Furthermore, a randomized clinical trial conducted by Pessanha et al. demonstrated the effects of a CMC hydrogel loaded with epidermal growth factor (EGF) on biofilm formation in the wounds of patients with DM [66]. Despite no differences in overall bacterial loads and virulense genes, they revealed that the loaded hydrogels were colonized by bacterial strains that presented lower biofilm formation capacities [66].

#### 3.1.3. Chitosan

Chitosan is a natural biomaterial composed of two linear sugar monomers: β(1,4)-linkedD-glucosamine and *N*-acetyl-D-glucosamine [24,80,93,94,95]. It is obtained by the alkaline deacetylation of chitin, the main component of the exoskeleton of crustaceans [24,80,93,94,95]. The type of chitosan, length of the chain, and degree of deacetylation influence its properties as a wound dressing [80,94,95]. Chitosan is quite unique, as it is the only known cationic polysaccharide in nature. Furthermore, chitosan presents several outstanding characteristics favorable for biomedical applications, including high biocompatibility, biodegradability, bioadhesivity, hydrophilicity, and hemostatic and antimicrobial properties [60,80,93,94,95,96,97,98]. Nevertheless, it exhibits some weaknesses such as chemical characteristics that limit its flexibility and consequent clinical use, although these weaknesses are preventable through chemical modifications and cross-linking with other materials [61]. A good example is the introduction of tannic acid (TA), with its excellent ROS reducing capacity and hemostatic activity, into a quaternized chitosan matrix by Pan et al. [67]. Quaternized chitosan is known to exhibit better water solubility and antibacterial properties compared to chitosan [99,100]. Accordingly, this modified chitosan hydrogel showed a decrease in ROS, a hemostatic effect, and *S. aureus* and *E. coli* inhibition in vitro; in addition, it fostered coagulation, decreased inflammation, and improved collagen deposition in full-thickness wounds of diabetic rats [67]. Zhou et al. also used quaternized chitin (QC) and TA to form QC/TA layer-by-layer-deposited TA/Ag-modified polylactic acid (PLA)/polyurethane (PU) hybrid nanofibers [68]. The formed hybrid nanofibers showed great flexibility, antibacterial activity against *S. aureus* and *E. coli*, and a reduction in ROS in vitro [68]. Moreover, Lee et al. fabricated composite hydrogels made of chitosan and polyvinyl alcohol (PVA) for loading of polyhexamethylene biguanide (PHMB), perfluorocarbon nanoemulsions, and chitosan nanoparticles loaded with EGF [69]. This composite hydrogel exhibited the sustained release of PHMB and EGF, promoting antibacterial activity against *S. aureus* and *S. epidermidis*, and the cell growth of human KERTr keratinocytes, essential for wound repair [69]. In addition, the produced hydrogels stimulated re-epithelialization, improved collagen deposition and maturation, and reduced inflammatory responses in full-thickness wounds of diabetic rats [69].

#### 3.1.4. Collagen

Collagen is a fibrous protein composed of long polypeptide chains normally produced by fibroblasts in mammals, and represents the major component of the extracellular matrix [19,21,22,87,101]. To date, 29 types of collagen have been identified, with collagen type I being the most abundant [19,102]. Its high biocompatibility and biodegradability, low immunogenicity, and ability to ensure cell attachment make collagen a suitable option for therapeutic applications [19,60,101,102,103]. Despite a very low antigenicity being induced, this can be controlled through the removal of the terminal telopeptide from collagen molecules [19]. Moreover, collagen can modulate cells to boost new collagen deposition and organization [101,102]. However, the low swelling capability and mechanical strength and the high degradation rate may also be limitations for certain purposes, although it is possible to overcome these limitations through chemical modifications or cross-linking with other materials [101,103]. Ma et al. used porcine small intestinal submucosa (SIS), mainly composed of collagen, to create a functional hydrogel for the loading of umbilical cord mesenchymal stem cell (ucMSC)-derived small extracellular vesicles and fusion peptides [70]. SIS was modified through catecholamine chemistry, in order to enhance the adhesion ability and biomechanical properties of the hydrogel [70]. This modified collagen dressing exhibited good mechanical strength, and NIH-3T3 fibroblast proliferation, migration and adhesion enhancement, as well as tube formation in EA.hy926 vascular endothelial cells [70]. The healing of full-thickness wounds in diabetic rats was also demonstrated [70]. Another study employed a blend-electrospinning process for the fabrication of electrospun nanofibers made of chitosan, PEO, and collagen [71]. These electrospun nanofibers were used for the sustained release of up to 3 days of curcumin (Cur), a strong anti-inflammatory and anti-infective agent, without causing toxicity towards human dermal fibroblasts [71]. Moreover, the Cur-loaded electrospun nanofibers significantly promoted the wound area closure of full-thickness wounds in non-diabetic rats [71].

#### 3.1.5. Dextran

Dextran, a hydrophilic polysaccharide produced by bacteria, consists of linear chains of α-1,6-linked d-glucopyranose residues [104,105]. It is known for its good biocompatibility, biodegradability, water retention, and easy modification, as well as its ability to reduce ROS and excess platelet activation, hence decreasing inflammatory responses and vascular thrombosis [72,104,106]. However, poor antibacterial activity has been linked to dextran, which requires cross-linking with other materials or loading with bioactive agents to handle this limitation. For example, Wei et al. engineered a composite hydrogel made of oxidized dextran and HA for the controlled release of an antimicrobial peptide (AMP) and platelet-rich plasma (PRP) [72]. This composite hydrogel exhibited antibacterial activity against *S. aureus* and *P. aeruginosa*, anti-inflammatory activity, and collagen deposition and angiogenesis properties, thus improving the healing of infected wounds in diabetic mice [72]. Guo et al. also demonstrated the potential of mixing ethylenediamine-modified gelatin with oxidized dextran for the loading of zinc oxide nanoparticles (ZnO-NPs) with antibacterial activity and low pH responsiveness, and paeoniflorin (Pf) with inherent angiogenic activity and ROS responsiveness [73]. This composite hydrogel achieved sequential hemostatic, antibacterial, and angiogenic activities, thus promoting the healing of chronically *S. aureus*-infected wounds in diabetic rats [73]. Wu et al. further showed the potential of combining oxidized dextran and the antimicrobial peptide DP7 to form a dual-function pH-sensitive hydrogel for the loading of ceftazidime (CAZ), an antibiotic [74]. This hydrogel was proved to exhibit a synergistic action able to eradicate multi-drug-resistant (MDR) bacteria such as *P. aeruginosa* and to induce the scarless healing of *P. aeruginosa*-infected wounds in diabetic mice [74].

#### 3.1.6. Fibrin

Fibrin is a natural polymeric material formed in the body during the first phase of the healing process when fibrinogen is activated [21,107]. It acts as a plug to cover the injured endothelium and consequently stop the bleeding [21,107]. In addition, fibrin may behave as a scaffold for leukocytes and endothelial cells throughout tissue regeneration [107]. Recently, fibrin has gained particular attention as a biomaterial for its bulk stiffness, degradability, and suitable porosity, as well as its role in promoting wound healing [75,107]. In comparison to dressings made of collagen found in more mature tissues, fibrin dressings may present a rapid degradation rate and a more porous structure with smaller typical periodic distances, due to its smaller length [108,109]. Nilforoushzadeh et al. demonstrated how cross-linking with collagen further boosted fibrin characteristics, by improving its major weakness: its poor mechanical and elastic properties [75]. The fibrin–collagen hydrogels were then used to encapsulate stromal vascular fraction (SVF) cells for the enhancement of healing in patients with hard-to-heal diabetic wounds [75].

#### 3.1.7. Gelatin

Gelatin is a natural polymeric material obtained from partial hydrolysis of insoluble collagen type I [19,110]. As a collagen derivative, it shares many of its characteristics, i.e., great biocompatibility, flexibility, stability, hydrophilicity, non-immunogenicity, and biodegradability [19]. As a smaller polypeptide, gelatin exhibits a smooth and viscoelastic material profile that contrasts with the more rigid and robust characteristics of collagen [19]. Moreover, gelatin is readily obtained from cost-effective resources, such as porcine skin, making it a highly attractive biomaterial for a diverse range of biomedical applications [19]. Although gelatin’s mechanical properties may be considered limiting in certain applications, typically these limitations have been mitigated through cross-linking with other biomaterials [19,110,111]. In addition, gelatin has also been shown to present hemostatic activity that is suitable to initiate the wound healing process and to absorb wound exudates, thus creating an appropriate microenvironment for the next phases of wound healing [19]. For example, recent studies have explored the potential of methacrylated gelatin (GelMA) shape-memorable cryogels for the controlled release of endothelial progenitor cells (EPCs) and acid fibroblast growth factor (aFGF) [76]. The incorporation of methacrylate groups enables the visible-light-induced cross-linking of gelatin into a stable hydrogel, while preserving its intrinsic enzymatic degradability [76]. Moreover, the methacrylated gelatin cryogel exhibited enhanced pressure ulcer healing in a diabetic rat model, due to the upregulation of HIF-1α at the wound site [76]. Other authors developed gelatin microspheres loaded with adipose-derived stem cells (ADSC) [77]. These ADSC-loaded gelatin microspheres were capable of accelerating the healing of full-thickness wounds in diabetic rats, through the promotion of M2 macrophage polarization, collagen deposition, angiogenesis, and hair follicle formation [77]. As previously described, Guo et al. revealed the potential of mixing ethylenediamine-modified gelatin with oxidized dextran for the loading of ZnO-NPs (with antibacterial activity) and Pf (with angiogenic activity) [73]. The formed hydrogels were shown to achieve sequential hemostatic, antibacterial, and angiogenic activities in response to pH and ROS variations, consequently promoting the healing of chronically *S. aureus*-infected wounds in diabetic rats [73].

#### 3.1.8. Hyaluronic Acid

HA is a linear polysaccharide found in the extracellular matrix of connective tissues in vertebrates and even in bacteria [80,87]. As a non-sulfated anionic glycosaminoglycan, HA displays good biocompatibility, biodegradability, and gel-forming properties, making it widely used in biomedical applications [80,87]. Moreover, HA plays a relevant role in inflammation, angiogenesis, and subsequent wound healing [60,61]. Indeed, high-molecular-weight HA may interact with the plasma membrane receptors of cells, such as CD44 receptors, for the enhancement of capillary formation [60,61]. In addition, HA may also induce inflammatory cells to eliminate invading microorganisms, and encourage fibroblasts and keratinocytes to migrate and proliferate to the wound bed through the regulation of pro-inflammatory cytokine synthesis, thus fostering wound healing [60,61]. However, HA also presents some drawbacks such as poor mechanical properties and structural stability, high water solubility, and no antimicrobial activity, therefore limiting its clinical use [60,80]. Nevertheless, studies have shown that some of thdse limitations can be bypassed by formulating composite hydrogels made of oxidized dextran and HA [72]. This composite hydrogel with the controlled release of an AMP and PRP exhibited antibacterial activity against *S. aureus* and *P. aeruginosa*, anti-inflammatory activity, and collagen deposition and angiogenesis properties, thus improving the healing of infected wounds in diabetic mice [72]. In turn, Xiong et al. developed HA hydrogels with integrated manganese dioxide (MnO_2_) nanoenzymes, which can decompose endogenous ROS, i.e., hydrogen peroxide, into oxygen, for the timed release of M2 macrophage-derived exosome (M2 Exos) and fibroblast growth factor 2 (FGF-2) [78]. This HA-based hydrogel resulted in the improved healing of wounds in diabetic mice, through the eradication of bacterial infection, diminution of oxidative stress, supply of oxygen, and stimulation of angiogenesis and epithelialization [78]. Moreover, Xu et al. designed a hybrid hydrogel made of phenylboronic acid (PBA) with glucose sensitivity modified onto a HA chain, and polyethylene glycol diacrylates (PEG-DA), for the release of myricetin (MY), a molecule with strong antioxidant activity [79]. This glucose-responsive hybrid PEG-DA/HA-PBA hydrogel exhibited a glucose-triggered release of MY, and an efficient elimination of ROS, as well as an ameliorated inflammatory response, angiogenesis, and the tissue remodeling of full-thickness wounds in diabetic rats [79].

### 3.2. Synthetic Materials

Synthetic materials offer advantages over naturally occuring materials, such as the absence of lot-to-lot variability and the ability to fine-tune and predict mechanical properties. However, synthetic materials do have some drawbacks that can limit their clinical application. These disadvantages include lower bioactivity and biodegradability, reduced capacity to mimic native tissue structure and function, and a greater likelihood of triggering inflammatory responses [24,60]. Indeed, both natural biomaterials and synthetic materials possess advantages and disadvantages, underlining the importance of appropriate material selection and the potential of natural/synthetic material combinations for the development of wound dressings [112]. It is important to note that cross-linking between natural and synthetic materials can also weaken some biological and wound healing activities, reducing their potential as chronic wound dressings [23]. This can be overcome with the loading of bioactive agents in the wound dressings to achieve the enhanced healing of chronic wounds such as DFUs. Common synthetic materials include polycaprolactone (PCL), polyethylene glycol (PEG), polyethylene oxide (PEO), polylactic acid (PLA), poly(lactic-co-glycolic acid) (PLGA), polyvinyl alcohol (PVA), and polyvinyl pyrrolidone (PVP). Synthetic materials that have been recently evaluated in research settings as promising wound dressings and their respective formulation and main properties are summarized in Table 2.

#### 3.2.1. Polycaprolactone

PCL is a synthetic polyester known for its excellent mechanical strength and consequent stability [127,128,129]. It can be obtained from low-cost resources via the ring-opening polymerization of ε-caprolactone monomers using a wide range of catalysts, and exhibits good biocompatibility [87,127,128,130]. However, PCL’s semicrystalline and hydrophobic nature results in a slow degradation rate, and poor wettability, cell attachment, and tissue integration, thus limiting its clinical use as a wound dressing [129,130]. In addition, it may also lack biological activity [129,130]. To overcome some of these issues, researchers have combined PCL with other materials and have resorted to loading with bioactive agents [127,128]. For example, Jiang et al. developed composite poly(D,L-lactic acid) (PDLLA)/PCL electrospun scaffolds to achieve better mechanical stability under wet conditions [113]. These composite scaffolds were then nanocoated with amorphous calcium phosphate (ACP) and silicon ion (Si^4+^) to further improve cell proliferation and migration, and to encourage their angiogenesis capacities [113]. Coated nanofibrous scaffolds enhanced the healing of full-thickness wounds in diabetic mice by stimulating angiogenesis, collagen deposition, and re-epithelialization [113]. In turn, Cam et al. designed composite nanofibrous scaffolds made of chitosan, gelatin, PCL, and PVP for the combined loading of pioglitazone (PHR) and metformin (MET), oral antidiabetics that also display anti-inflammatory activities [114]. These composite nanofibrous scaffolds demonstrated increased wettability and hydrophilicity, as well as high tensile strength and cytocompatibility with L929 fibroblasts [114]. In addition, loaded composite scaffolds showed the accelerated healing of full-thickness wounds in diabetic rats by improving collagen remodeling, re-epithelialization, and hair follicle formation, while lowering pro-inflammatory tumor necrosis factor alpha (TNF-α), interleukin-6 (IL-6), interleukin-1 beta (IL-1β), and nuclear factor kappa light chain enhancer of activated B cells (NF-κB) levels [114].

#### 3.2.2. Polyethylene Glycol

PEG is an amphiphilic polymer known for its excellent biocompatibility, biodegradability, stability, and low-cost preparation [22,60,101]. Nonetheless, the preparation of wound dressings made of this synthetic polymeric material commonly requires the use of cross-linking agents such as formaldehyde that could turn them cytotoxic [60]. Therefore, developing novel strategies to reduce the toxicity and enhance the biological activity of PEG-based dressings is thus imperative. One effective option is the cross-linking of PEG with chitosan to form hydrogels for the loading of silver nanoparticles (Ag-NPs) that own attractive antimicrobial, anti-oxidant, anti-inflammatory, and anti-platelet properties [115,131]. These loaded chitosan-PEG hydrogels showed high porosity and a high degree of swelling, as well as improved antioxidant capacity and antimicrobial activity against *P. aeruginosa*, *E. coli*, and *S. aureus* [115]. In addition, loaded hydrogels boosted the healing of wounds in diabetic rabbits [115]. Wu et al. similarly combined four-armed aldehyde-terminated PEG (4-arm PEG-CHO) with quaternized chitosan to form a hydrogel for the loading of DFO [116]. The formed hydrogels displayed good mechanical properties and biocompatibility, as well as improved angiogenesis and the accelerated healing of *S. aureus*-infected wounds in diabetic rats [116]. Furthermore, Xu et al. developed PDLLA-PEG-PDLLA hydrogels for the loading of Prussian blue nanoparticles (PBNPs) with great ROS-scavenging capacity [117]. These PBNP-loaded hydrogels stimulated angiogenesis, and decreased ROS and pro-inflammatory cytokine (IL-6 and TNF-α) production, thus favoring the healing of full-thickness wounds in diabetic mice [117].

#### 3.2.3. Polyethylene Oxide

PEO is a synthetic polymer with a similar structure to PEG, but that typically displays higher molecular weights [132,133]. It is a neutral polymer that can be obtained through the polymerization of ethylene oxide using a metallic catalyst [133,134]. PEO is a non-ionic polymer that exhibits good biocompatibility, hydrophilicity, and biodegradability [133,134,135]. Moreover, its high-molecular-weight grade contributes to high viscosity, which is a suitable characteristic for forming strong and solid gels favorable to be applied as transdermal dressings [135]. Despite its poor biological activity, PEO has been combined with other materials for the enhancement of intrinsic properties and subsequent biomedical application [92]. As previously described, Azarniya et al. developed composite scaffolds consisting of nanofibrous mats made of bacterial cellulose and PEO-modified keratin, as well as TG-conjugated hydrogels [65]. PEO was used as an additive to enhance mechanical properties such as the spinnability of pure keratin. The modified nanofibers with TG-conjugated hydrogels showed superior overall mechanical properties such as hydrophilicity, elasticity, tensile strength, and ductility [65]. In addition, good biocompatibility with L929 fibroblasts was observed, as well as enhanced cell adhesion and proliferation, thus underlining the potential of this hybrid composite scaffold as an attractive wound dressing [65]. As mentioned earlier, Jirofti et al. employed a blend-electrospinning process for the fabrication of electrospun nanofibers made of chitosan, PEO, and collagen [71]. Here, PEO was used as an additive to enhance mechanical properties such as the spinnability of chitosan. These electrospun nanofibers were used for the sustained release of up to 3 days of Cur, a strong anti-inflammatory and anti-infective agent, without causing toxicity towards human dermal fibroblasts [71]. Moreover, the loaded electrospun nanofibers revealed significant wound area closure of full-thickness wounds in non-diabetic rats [71].

#### 3.2.4. Polylactic Acid

PLA is an aliphatic polyester that is easily obtained in an eco-friendly way through the condensation of lactic acid, followed by the ring-opening polymerization of cyclic lactides [68,118,136,137]. Its good biocompatibility, biodegradability, and relatively high mechanical properties make PLA an appealing material for biomedical applications [68,118,119]. In addition, it has been associated with enhanced cell proliferation and growth factor signaling due to its inherent characteristics that make it similar to natural collagen fibers in the extracellular matrix [119]. A limitation to the clinical use of PLA is its low hydrophilicity, which can be mitigated by combining it with other materials or loading it with bioactive agents [118]. For instance, Yu et al. developed porous PLA nanofiber membranes that were further decorated with sulfated chitosan (SCS) and polydopamine-gentamicin (PDA-GS) [118]. The combination with SCS was used to improve the hydrophilicity of PLA nanofibers and enhance M2 macrophage polarization, while PDA and GS were used to improve anti-inflammatory and anti-bacterial activities [118]. Overall, these PLA/SCS/PDA-GS nanofiber membranes showed the immunomodulation of M2 macrophage polarization and stimulation of VEGF secretion in Raw 264.7 macrophages [118]. In addition, developed nanofiber membranes exhibited antibacterial activity against *S. aureus*, further highlighting their potential as attractive wound dressings for diabetic wound healing [118]. Other authors designed multilayered nanofibrous PLA patches for the loading of phenytoin (PHN), sildenafil citrate (SILD), and simvastatin (SIM) with anti-inflammatory, angiogenic, and lymphangiogenic properties, respectively [119]. Loaded patches kept their physicochemical and mechanical properties, as well as biocompatibility and cell adhesion capacity, in human dermal fibroblasts [119]. In addition, the developed nanofibers showed adequate fibroblast proliferation, angiogenesis, and lymphangiogenesis, resulting in a proper and scarless healing of full-thickness wounds in diabetic rats [119]. Furthermore, Di Cristo et al. produced co-electrospun fibers made of PLA and PVP for the loading of quercetin (QUE), a bioactive molecule with high anti-inflammatory, anti-oxidant, antimicrobial, and wound healing properties [120]. The prepared fibers exhibited good hydrophilicity, and a fast initial release of QUE that was followed by a continuous and constant release for up to 120 h [120]. Moreover, these nanofibers showed great antibiofilm activity against *S. aureus*, and anti-inflammatory potential in PMA-differentiated THP-1 macrophages, making them hopeful alternatives for the effective management of diabetic foot infections [120].

#### 3.2.5. Poly(lactic-co-glycolic acid)

PLGA is a linear co-polymer resulting from the ring-opening polymerization of lactide and glycolide [138]. This aliphatic co-polymer displays excellent biocompatibility and mechanical properties, such as mechanical strength and flexibility, as well as low hydrophobicity and crystallinity compared to PLA, leading to a faster biodegradation rate [87,138]. Moreover, its mechanical properties and degradation rate are easily controllable and tunable by modifying the ratio of PLA to polyglycolic acid (PGA), further highlighting its potential as a wound dressing [138]. However, the relatively high cost of PLGA can pose a challenge, restricting its use in biomedical applications [138]. Nonetheless, Zheng et al. still employed PLGA to form composite nanofiber membranes with cellulose nanocrystals (CNC) for the loading of neurotensin (NT), an inflammatory modulator [121]. This composite nanofiber membrane exhibited the sustained release of NT for up to two weeks, and also demonstrated good cytocompatibility and fibroblast adhesion, in addition to the spreading and proliferation stimulation of 3T3 fibroblasts [139]. In addition, the NT-loaded PLGA/CNC composite nanofiber membranes showed accelerated healing of full-thickness wounds in diabetic mice, with better epidermal and dermal regeneration scores, and decreased pro-inflammatory cytokine expression (IL-1β and IL-6) [121]. In turn, Hasan et al. developed PLGA nanoparticles decorated with polyethylenimine/diazeniumdiolate (PEI/NONOate), possessing the ability to bind to the bacteria biofilm matrix [122]. The formed NPs were used for the extended release of up to 4 days of nitric oxide (NO), a bioactive agent with antibiofilm and wound healing activities, yet with a short half-life and limited diffusion [122]. The resulting loaded NPs showed enhanced antibiofilm activity, through a strong binding to the methicillin-resistant *S. aureus* (MRSA) biofilm matrix [122]. Furthermore, PLGA-PEI/NO NPs improved the healing of MRSA biofilm-infected wounds in diabetic mice, with complete bacterial biofilm eradication [122].

#### 3.2.6. Polyvinyl Alcohol

PVA is a synthetic resin derived from the hydrolysis of polyvinyl acetate in an alcohol solution, followed by treatment with an alkaline catalyst [21,87]. This synthetic linear polymer displays good biocompatibility, biodegradability, hydrophilicity, and semi-crystalline features [21,22,60,87]. PVA also possesses good mechanical properties such as swelling capacity, tensile strength, and chemo-thermal stability, despite a lack of elasticity [22,60,87,110]. However, PVA exhibits diminished hemostatic, bioadhesive, and antimicrobial activities [60,101,110]. To address these limitations, PVA-based dressings can be formulated with other materials or loaded with bioactive agents. For example, Lv et al. combined chitosan (CS) with PVA to form electrospun nanofibers to be loaded with ursolic acid (UA), which has the ability to lower blood glucose, and anti-oxidant and anti-inflammatory characteristics [123]. CS/PVA nanofibers loaded with 0.2% (*w*/*v*) of UA showed good hydrophilicity and wettability, as well as the sustained and non-toxic release of UA [123]. Furthermore, the loaded nanofibers enhanced the stimulation of M2 macrophage polarization, decreased pro-inflammatory TNF-α and IL-6 levels, and reduced ROS in Raw 264.7 macrophages [123]. Finally, CS/PVA/UA nanofibers accelerated the closure of full-thickness wounds in diabetic mice, with enhanced revascularization and re-epithelialization, increased collagen deposition and remodeling, and improved hair follicle regeneration [123]. In turn, Ningrum et al. used the freeze–thaw process to develop hydrogels composed of PVA and graphene oxide (GO) for the loading of Moringa oleifera leaf (MOL) extract, with antimicrobial and anti-inflammatory activities [124]. The combination with GO further increased the tensile strength of the loaded hydrogels [124]. Overall, PVA/MOL/GO hydrogels demonstrated a high water content and equilibrium swelling ratio, as well as good cytocompatibility with 3T3-L1 fibroblasts [124]. In addition, loaded hydrogels showed great antibacterial activity against *S. aureus* and *E. coli*, although a lower inhibition was observed against *E. coli* [124]. Lastly, PVA/MOL/GO hydrogels greatly stimulated the migration of 3T3L1 fibroblasts after 6 h, thus emphasizing their potential for wound healing [124].

#### 3.2.7. Polyvinyl Pyrrolidone

PVP, or povidone, is an amphiphilic polymer synthesized through the radical polymerization of N-vinylpyrrolidone monomer [140,141]. This synthetic polymer exhibits favorable biocompatibility, biodegradability, wettability, and chemical stability [60,87,141]. In addition, PVP has bioadhesive and antibacterial (mainly against Gram-negative bacteria) properties, useful characteristics for wound dressings [21,60]. However, to correspond to the current needs of biomedical therapeutics, the combination of PVP with other materials to further improve its characteristics has been widely reported in the literature. As previously described, Cam et al. designed composite nanofibrous scaffolds made of chitosan, gelatin, PCL, and PVP for the combined loading of pioglitazone (PHR) and metformin (MET), oral antidiabetics that also display anti-inflammatory activities [114]. These composite nanofibrous scaffolds demonstrated increased wettability and hydrophilicity, as well as high tensile strength and cytocompatibility with L929 fibroblasts [114]. In addition, loaded composite scaffolds showed the accelerated healing of full-thickness wounds in diabetic rats by improving collagen remodeling, re-epithelialization, and hair follicle formation, while lowering pro-inflammatory TNF-α, IL-6, IL-1β, and NF-κB levels [114]. Other authors developed a bilayered film patch composed of PVP and HA for the loading of the antiseptic Neomercurocromo^®^ (Neo), and the antibiotic ciprofloxacin (Cipro) [125]. PVP/HA-based bilayer film patches displayed good self-adhering strength, the sustained release of the bioactive agents for up to 5 days, and excellent antibacterial activity against *S. aureus*, *E. coli*, and *P. aeruginosa* [142]. In addition, these loaded patches decreased pro-inflammatory IL-6, IL-1β, and TNF-α levels, and enhanced the healing of full-thickness wounds in diabetic mice [125]. Furthermore, Yu et al. grafted PVP-iodine (PVPI) onto CMCS microspheres for the further improvement of chitosan characteristics [126]. These composite microspheres promoted antibacterial activity against *S. aureus* and wound healing in diabetic mice [126].

## 4. Biomaterial-Based Wound Dressings in the Clinical Setting

The use of biomaterials as wound dressings for DFUs has been widely explored in research settings, but not many have yet been tested in the clinical setting, and even fewer have been approved and have become commercially available specifically for DFU management over the last five years. Indeed, there are several barriers that delay the clinical translation of wound dressings from the bench to the market. On the one hand, the performance of chemical modifications on biomaterials to further improve their intrinsic characteristics requires supplementary validation by the U.S. Food and Drug Administration and/or the European Union Medical Device Regulation [143]. This results in an additional step for material safety and quality assessment, prior to the time-consuming technology validation process itself. On the other hand, common techniques to characterize traditional wound dressings may not be adequate to prove superior capabilities in the wound healing enhancement of the most innovative wound dressings and may require more accurate models of the DFU condition and more complete techniques to simultaneously evaluate the tissue regeneration rate, drug release effectiveness, and stimuli-responsive mechanisms [143,144]. Furthermore, innovative wound dressings often possess a multicomponent and multifunctional core, leading to difficult device classification and validation processes. Undeniably, it is often ambiguous to determine which property is the primary mode of action of wound dressing for adequate device classification, owing to the combined mode of action of numerous wound dressings [143]. If the primary mode of action is drug delivery, the wound dressing is classified as a drug, whilst if the primary mode of action is not drug delivery, it is classified as a medical device [145]. Thus, most of these wound dressings should undergo both drug and device validation. Finally, the cost/benefit ratio is often unattractive for healthcare systems, thus limiting the access to cutting-edge therapeutic approaches for patients with chronic DFUs [146]. A list of the most recent clinical trials involving biomaterial-based wound dressings for DFU treatment and their respective formulations is presented in Table 3.

On the one hand, many examples are based on natural biomaterials due to their excellent biocompatibility, biodegradability, and low antigenicity, as well as their ability to mimic native tissue structure and function, thus fostering suitable wound healing. For instance, a phase I trial evaluated the potential of a collagen scaffold loaded with mesenchymal stromal cells to enhance cell proliferation and migration and subsequent wound healing [147]. In turn, two phase III trials assessed the safety and effectiveness of a fibrin glue loaded with PRP, which formed a protective barrier against the external environment and fostered re-epithelialization [148,149], and Granexin^®^, a hydroxyethyl cellulose gel for the loading of aCT1, which is a synthetic peptide known to stimulate re-epithelialization and consequent wound closure [150,151]. A phase III trial evaluated the potential of an allogeneic ADSC-loaded fibrin gel that possesses anti-inflammatory and pro-angiogenic effects and can stimulate granulation tissue formation [152,153]. In addition, many pilot trials have emerged as small-scale preliminary studies to evaluate the feasibility and assist the planning and modification of larger-scale studies for the efficacy assessment of wound dressings such as PRP Concepts Fibrin Bio-Matrix, a fibrin-based matrix enriched with platelets [154]; Piscean-derived collagen dressing with hemostatic effects [155]; Omeza, a fish-derived collagen matrix that maintains a moist healing environment to promote wound healing [156]; Helaquis Matrix, a HA matrix that facilitates cell migration and proliferation and maintains wound moisture [157]; and Meso Wound Matrix™, which is another collagen matrix but derived from porcine peritoneum membrane [158,159]. Furthermore, a pivotal study, which is intended to demonstrate and confirm safety and efficacy and to estimate the incidence of common adverse effects, further assessed the potential of NanoSALV catalytic, an antimicrobial wound dressing gel made of CMC that regulates wound moisture [160].

On the other hand, examples based on synthetic materials have also emerged due to their good mechanical properties, as well as their broad variety. For instance, many pilot trials have emerged that are focused on the efficacy assessment of wound dressings such as Exufiber Ag^+^, a PVA fiber which has great exudate absorption capacity, and antimicrobial and anti-oxidant effects due to Ag^+^ [161]; and PHOENIX Wound Matrix^®^, which consists of a PCL-based matrix that mimics the native ECM and handles the chronicity and persistent inflammation of chronic wounds [162]. Furthermore, some clinical trials are investigating the potential of combining natural and synthetic materials such as Fitostimoline^®^, which consists of a hydroxyethyl cellulose and PEG-based hydrogel that form a protective barrier against the external environment to foster proper wound healing [163].

Another example is represented by the Post Market Clinical Follow-up (PMCF) trials that are still ongoing to further assess the safety and performance of the CE-marked Chitocare^®^ and Cutimed^®^ [164,165]. Chitocare^®^ consists of a wound healing gel, which owes its wound healing and anti-infective effects to chitosan [164], while Cutimed^®^ is a gelling fiber made of sodium CMC and high-density polyethylene and polyethylene terephthalate, allowing the absorption of wound exudates and subsequent conversion into a soft gel that maintains a moist environment to support wound healing [165].

## 5. Conclusions and Future Perspectives

In recent years, there has been a wealth of research focused on biomaterials and their inherent properties as wound dressings for chronic DFUs. However, further investigation remains necessary to effectively address the challenging macro and micro complexity of the wound environment of DFUs. Indeed, only a few biomaterial-based therapeutic strategies have successfully reached translation to the market, despite numerous materials being explored in pre- and clinical trials. As a result, DFUs continue to pose a significant threat to the well-being of individuals with DM and place a burden on the healthcare system while waiting for truly effective therapeutic strategies. Therefore, to bridge the gap between research and clinical application, a combined and collaborative effort among multidisciplinary teams is essential to develop more effective treatment options. In this approach, key factors to consider include material selection, chemical modifications, combination with other materials, and loading with biomolecules possessing pro-angiogenic, anti-inflammatory, and antimicrobial properties. Furthermore, more accurate models of non-healing DFUs need to be included in future research to efficiently prove the efficacy of novel biomaterial-based therapeutic approaches. This should include chronic wound models with polymicrobial infections and even biofilms, in order to better mimic the DFU condition. Moreover, the referral to multidisciplinary teams must remain a key point for the optimal management of DFUs. This should include adjuvant health and well-being areas such as dietary counseling and stress-reducing therapies. Indeed, a more holistic personalized approach should undeniably be part of DFU management. Addressing all of these challenges could thus potentially increase clinical trial success rates, ultimately leading to the development of effective treatment approaches for polymicrobial-infected chronic wounds such as DFUs.

## Figures and Tables

**Table 1:** Natural biomaterials with cutting-edge potential as wound dressings and their respective main properties.

Biomaterial	Structural Formula	Formulation	Main Properties/Outcomes	Ref.
Alginate	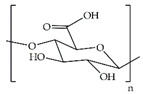	Patches filled with sodium alginate gel beads containing active *S. elongatus*	More efficient delivery of dissolved oxygen Improved chronic wound healing and skin graft survival in diabetic mice	[62]
		Calcium ion cross-linked sodium alginate hydrogels for loading of DFO and Cu-NPs	Increased HIF-1α and VEGF levels Enhanced angiogenesis, decreased chronic inflammation, and accelerated wound healing in diabetic mice	[63]
		CMCS cross-linked sodium alginate hydrogels for loading of Ir-fliq	Improved healing of *S. aureus*-infected chronic wounds in diabetic mice, by inhibition of bacterial growth through PACT	[64]
Cellulose	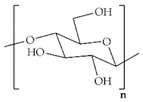	Composite scaffolds consisting of nanofibers made of cellulose and PEO-modified keratin, and TG-conjugated hydrogels	Enhanced mechanical properties such as hydrophilicity, elasticity, tensile strength, and ductility Good biocompatibility with L929 fibroblasts, as well as enhanced cell adhesion and proliferation	[65]
		CMC hydrogels for loading of EGF *	No differences in overall bacterial loads and virulence genes, but colonization by bacterial strains with lower biofilm formation capacities	[66]
Chitosan	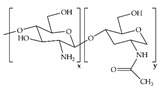	Quaternized chitosan/TA hydrogels	Decreased ROS, hemostatic effect, and *S. aureus* and *E. coli* inhibition in vitro Fostered coagulation, decreased inflammation, and improved collagen deposition in full-thickness wounds of diabetic rats	[67]
		QC/TA layer-by-layer-deposited TA/Ag-modified PLA/PU hybrid nanofibers	Great flexibility Antibacterial activity against *S. aureus* and *E. coli* Decreased ROS in vitro	[68]
		Composite hydrogels made of chitosan and PVA for loading of PHMB, perfluorocarbon nanoemulsions, and chitosan nanoparticles loaded with EGF	Sustained release of PHMB and EGF, promoting antibacterial activity against *S. aureus* and *S. epidermidis*, and cell growth of KERTr cells Enhanced re-epithelialization, improved collagen deposition and maturation, and decreased inflammatory responses in full-thickness wounds of diabetic rats	[69]
Collagen	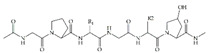 (collagen type I)	Porcine collagen hydrogel for loading of ucMSC-derived small extracellular vesicles and fusion peptides	Good mechanical strength Enhanced NIH-3T3 fibroblast proliferation, migration and adhesion Enhanced tube formation in EA.hy926 cells Improved healing of wounds in diabetic rats	[70]
		Electrospun nanofibers made of chitosan, PEO, and collagen for loading of Cur	Sustained release of Cur up to 3 days No toxicity towards human dermal fibroblasts Significant wound area closure of wounds in non-diabetic rats	[71]
Dextran	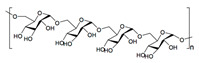	Oxidized dextran and HA composite hydrogels for loading of AMP and PRP	Antibacterial activity against *S. aureus* and *P. aeruginosa*, anti-inflammatory activity, collagen deposition and angiogenesis properties, and enhancing healing of infected wounds in diabetic mice	[72]
		Gelatin and oxidized dextran hydrogels for loading of ZnO-NPs and Pf	Sequential hemostatic, antibacterial, and angiogenic activities in infected wounds of diabetic rats	[73]
		Oxidized dextran and peptide DP7 hydrogels for loading of CAZ	Eradication of MDR bacteria such as *P. aeruginosa* Scarless wound healing in diabetic mice	[74]
Fibrin	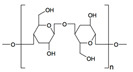	Collagen cross-linked fibrin hydrogels for loading of SVF cells **	Improved mechanical and elastic properties Enhanced healing activity in patients with hard-to-heal diabetic wounds	[75]
Gelatin		GelMA cryogels for loading of EPCs and aFGF	Improved stability and retained degradability Enhanced pressure ulcer healing in a diabetic rat model, due to upregulation of HIF-1α	[76]
		Gelatin microspheres for loading of ADSCs	Good biocompatibility and adaptive degradation rate Accelerated healing of wounds in diabetic rats	[77]
		Gelatin and oxidized dextran hydrogels for loading of ZnO-NPs and Pf	Sequential hemostatic, antibacterial, and angiogenic activities in *S. aureus*-infected wounds of diabetic rats	[73]
Hyaluronic acid (HA)	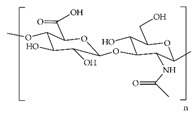	Oxidized dextran and HA composite hydrogel for loading of AMP and PRP	Antibacterial activity against *S. aureus* and *P. aeruginosa*, anti-inflammatory activity, and collagen deposition and angiogenesis properties, enhancing healing of infected wounds in diabetic mice	[72]
		HA hydrogels with integrated MnO_2_ nanoenzymes, for loading of M2 Exos and FGF-2	Improved healing of wounds in diabetic mice, through eradication of bacterial infection, diminution of oxidative stress, supply of oxygen, and stimulation of angiogenesis and epithelialization	[78]
		PEG-DA/HA-PBA hybrid hydrogels for loading of MY	Glucose-triggered release of MY Efficient elimination of ROS Ameliorated inflammatory response, angiogenesis, and tissue remodeling of wounds in diabetic rats	[79]

*^,^ ** Pilot clinical trials. DFO—deferoxamine; Cu-NPs—copper nanoparticles; HIF-1α—hypoxia-inducible factor 1 alpha; VEGF—vascular endothelial growth factor; CMCS—carboxymethyl chitosan; Ir-fliq—small molecular probe; PACT—photodynamic antimicrobial chemotherapy; PEO—polyethylene oxide; TG—tragacanth gum; CMC—carboxymethyl cellulose; EGF—epidermal growth factor; TA—tannic acid; ROS—reactive oxygen species; QC—quaternized chitin; Ag—silver; PLA—polylactic acid; PU—polyurethane; PVA—polyvinyl alcohol; PHMB—polyhexamethylene biguanide; ucMSC—umbilical cord mesenchymal stem cell; Cur—curcumin; AMP—antimicrobial peptide; PRP—platelet-rich plasma; ZnO-NPs—zinc nanoparticles; Pf—paeoniflorin; CAZ—ceftazidime; MDR—multi-drug-resistant; SVF—stromal vascular fraction; GelMA—methacrylated gelatin; EPC—endothelial progenitor cell; aFGF—ok; ADSC—acid fibroblast growth factor; MnO_2_—manganese dioxide; M2 Exos—M2 macrophage-derived exosome; FGF-2—fibroblast growth factor 2; PEG-DA—polyethylene glycol diacrylates; PBA—phenylboronic acid; MY—myricetin.

**Table 2:** Synthetic biomaterials with cutting-edge potential as wound dressings and their respective main properties.

Biomaterial	Structural Formula	Formulation	Main Properties/Outcomes	Ref.
Polycaprolactone (PCL)	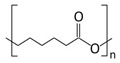	Composite PDLLA/PCL electrospun scaffolds nanocoated with ACP and Si^4+^	Better mechanical stability under wet conditions Enhanced healing of full-thickness wounds in diabetic mice, by stimulating angiogenesis, collagen deposition, and re-epithelialization	[113]
		Composite nanofibrous scaffolds made of chitosan, gelatin, PCL, and PVP for loading of PHR and MET	Increased wettability and hydrophilicity High tensile strength Cytocompatibility with L929 fibroblasts Accelerated healing of full-thickness wounds in diabetic rats by improving collagen remodeling, re-epithelialization, and hair follicle formation, while lowering pro-inflammatory TNF-α, IL-6, IL-1β, and NF-κB levels	[114]
Polyethylene glycol (PEG)	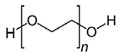	Chitosan cross-linked PEG hydrogels for loading of Ag-NPs	High porosity and degree of swelling Improved antioxidant capacity and antimicrobial activity against *P. aeruginosa*, *E. coli*, and *S. aureus* Boosted healing of wounds in diabetic rabbits	[115]
		Quaternized chitosan-combined 4-arm PEG-CHO hydrogels for loading of DFO	Good mechanical properties and biocompatibility Improved angiogenesis and accelerated healing of *S. aureus*-infected wounds in diabetic rats	[116]
		PDLLA-PEG-PDLLA hydrogels for loading of PBNPs	Enhanced angiogenesis, and decreased ROS, IL-6, and TNF-α production, thus favoring healing of full-thickness wounds in diabetic mice	[117]
Polyethylene oxide (PEO)	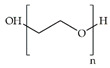	Composite scaffolds consisting of nanofibers made of cellulose and PEO-modified keratin, and TG-conjugated hydrogels	Enhanced mechanical properties such as hydrophilicity, elasticity, tensile strength, and ductility Good biocompatibility with L929 fibroblasts, as well as enhanced cell adhesion and proliferation	[65]
		Electrospun nanofibers made of chitosan, PEO, and collagen for loading of Cur	Sustained release of Cur for up to 3 days No toxicity towards human dermal fibroblasts Significant wound area closure of wounds in non-diabetic rats	[71]
Polylactic acid (PLA)	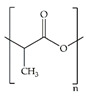	PLA/SCS/PDA-GS nanofiber membranes	Immunomodulation of M2 macrophage polarization and stimulation of VEGF secretion in Raw 264.7 cells Antibacterial activity against *S. aureus*	[118]
		Multilayered nanofibrous PLA patches for loading of PHN, SILD, and SIM	Great physicochemical and mechanical properties Good biocompatibility and cell adhesion capacity in human dermal fibroblasts Adequate fibroblasts proliferation, angiogenesis, and lymphangiogenesis, resulting in a proper and scarless healing of full-thickness wounds in diabetic rats	[119]
		Co-electrospun fibers made of PLA and PVP for loading of QUE	Good hydrophilicity Great antibiofilm activity against *S. aureus* Anti-inflammatory potential in PMA-differentiated THP-1 macrophages	[120]
Poly(lactic-co-glycolic acid) (PLGA)	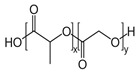	Composite PLGA/CNC nanofiber membranes for loading of NT	Sustained release of NT up to two weeks Good cytocompatibility and fibroblast adhesion, spreading and proliferation stimulation in 3T3 fibroblasts Accelerated healing of full-thickness wounds in diabetic mice, with better epidermal and dermal regeneration scores, and decreased IL-1β and IL-6 expression	[121]
		PLGA-PEI/NO NPs	Extended release for up to 4 days of NO Enhanced antibiofilm activity, through a strong binding to the MRSA biofilm matrix Improved healing of MRSA biofilm-infected wounds in diabetic mice	[122]
Polyvinyl alcohol (PVA)	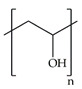	CS/PVA/UA nanofibers	Good hydrophilicity and wettability, as well as sustained and non-toxic release of UA Enhanced stimulation of M2 macrophage polarization, decreased pro-inflammatory TNF-α and IL-6 levels, and reduced ROS in Raw 264.7 cells Accelerated closure of full-thickness wounds in diabetic mice, with enhanced revascularization and re-epithelialization, increased collagen deposition and remodeling, and improved hair follicle regeneration	[123]
		PVA/MOL/GO hydrogels	Increased tensile strength High water content and equilibrium swelling ratio Good cytocompatibility in 3T3L1 fibroblasts Great antibacterial activity against *S. aureus* and *E. coli* Great migration of 3T3L1 fibroblasts after 6 h	[124]
Polyvinyl pyrrolidone (PVP)	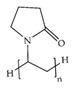	Composite nanofibrous scaffolds made of chitosan, gelatin, PCL, and PVP for loading of PHR and MET	Increased wettability and hydrophilicity High tensile strength Cytocompatibility with L929 fibroblasts Accelerated healing of full-thickness wounds in diabetic rats by improving collagen remodeling, re-epithelialization, and hair follicle formation, while lowering pro-inflammatory TNF-α, IL-6, IL-1β, and NF-κB levels	[114]
		PVP/HA-based bilayer film patches for loading of Neo and Cipro	Good self-adhering strength Sustained release of the bioactive agents for up to 5 days Antibacterial activity against *S. aureus*, *E. coli*, and *P. aeruginosa* Decreased IL-6, IL-1β, and TNF-α levels Enhanced wound healing in diabetic mice	[125]
		PVPI-grafted CMCS microspheres	Antibacterial activity against *S. aureus* Accelerated wound healing in diabetic mice	[126]

PDLLA—poly(D,L-lactic acid); ACP—amorphous calcium phosphate; Si^4+^—silicon ion; PHR—pioglitazone; MET—metformin; TNF-α—tumor necrosis factor alpha; IL-6—interleukin-6; IL-1β—interleukin-1 beta; NF-κB—nuclear factor kappa light chain enhancer of activated B cells; Ag-NPs—silver nanoparticles; CHO—aldehyde-terminated; DFO—deferoxamine; PBNP—Prussian blue nanoparticles; ROS—reactive oxygen species; TG—tragacanth gum; Cur—curcumin; SCS—sulfated chitosan; PDA—polydopamine; GS—gentamicin; VEGF—vascular endothelial growth factor; PHN—phenytoin; SILD—sildenafil citrate; SIM—simvastatin; QUE—quercetin; CNC—cellulose nanocrystals; NT—neurotensin; PEI—polyethylenimine; NO—nitric oxide; MRSA—methicillin-resistant *S. aureus*; CS—chitosan; UA—ursolic acid; MOL—*Moringa oleifera* leaf; GO—graphene oxide; Neo—Neomercurocromo^®^; Cipro—ciprofloxacin; PVPI—PVP-iodine; CMCS—carboxymethyl chitosan.

**Table 3:** Formulations of biomaterial-based wound dressings under clinical trials for DFU management. Search carried out on ClinicalTrials.gov from 2018 to 2023 (accessed on 19–21 April 2023).

Biomaterial	Formulation	Phase	ClinicalTrials.gov ID	Year
Collagen	Mesenchymal stromal cells in a collagen scaffold	I	NCT03509870	2018–2020
Fibrin	PRP-fibrin glue	III	NCT04315909	2019–2020
Hydroxyethyl cellulose	Granexin^®^—hydroxyethyl cellulose gel loaded with aCT1 peptide	III	NCT02667327	2020
Fibrin	Allogeneic ADSCs in a fibrin gel	II	NCT03865394	2019–2021
Fibrin	PRP Concepts Fibrin Bio-Matrix	Pilot	NCT02312596	2021
Hydroxyethyl cellulose and PEG	Fitostimoline^®^ hydrogel	IV	NCT05661474	2021–2022
Collagen	Piscean-derived collagen dressing	Pilot	NCT05324930	2021–2022
Collagen	Omeza collagen matrix	Pilot	NCT05417425	2022
HA	Hēlaquis Matrix—HA matrix	Pilot	NCT05198544	2022
PVA	Exufiber Ag^+^	Pilot	NCT05494450	2022
Collagen	Meso Wound Matrix™—porcine peritoneum membrane-derived collagen matrix	Pilot	NCT04182451	2019–2023
PCL	PHOENIX Wound Matrix^®^	Pilot	NCT04437537	2022–2023
CMC	NanoSALV catalytic antimicrobial wound dressing gel	Pivotal	NCT05619237	2022–2023
Chitosan	ChitoCare^®^	PMCF	NCT05570877	2022–2023
CMC and polyethylene	Cutimed^®^ gelling fiber	PMCF	NCT05148390	2022–2023

PRP—platelet-rich plasma; ADSC—adipose-derived stem cells; PEG—polyethylene glycol; HA—hyaluronic acid; PVA—polyvinyl alcohol; Ag^+^—silver cation; PCL—polycaprolactone; PMCF—Post Market Clinical Follow-up; CMC—carboxymethyl cellulose.

## Data Availability

Not applicable.

## References

[1] International Diabetes Federation (2021). IDF Diabetes Atlas 2021.

[2] Vibha S.P., Kulkarni M.M., Kirthinath Ballala A.B., Kamath A., Maiya G.A. (2018). Community based study to assess the prevalence of diabetic foot syndrome and associated risk factors among people with diabetes mellitus. BMC Endocr. Disord..

[3] Akkus G., Sert M. (2022). Diabetic foot ulcers: A devastating complication of diabetes mellitus continues non-stop in spite of new medical treatment modalities. World J. Diabetes.

[4] Brocco E., Ninkovic S., Marin M., Whisstock C., Bruseghin M., Boschetti G., Viti R., Forlini W., Volpe A. (2018). Diabetic foot management: Multidisciplinary approach for advanced lesion rescue. J. Cardiovasc. Surg..

[5] Da Silva J., Leal E.C., Carvalho E. (2021). Bioactive antimicrobial peptides as therapeutic agents for infected diabetic foot ulcers. Biomolecules.

[6] Hurlow J.J., Humphreys G.J., Bowling F.L., McBain A.J. (2018). Diabetic foot infection: A critical complication. Int. Wound J..

[7] Pouget C., Dunyach-Remy C., Pantel A., Schuldiner S., Sotto A., Lavigne J.P. (2020). Biofilms in diabetic foot ulcers: Significance and clinical relevance. Microorganisms.

[8] Edmonds M., Manu C., Vas P. (2021). The current burden of diabetic foot disease. J. Clin. Orthop. Trauma.

[9] Uivaraseanu B., Bungau S., Tit D.M., Fratila O., Rus M., Maghiar T.A., Maghiar O., Pantis C., Vesa C.M., Zaha D.C. (2020). Clinical, Pathological and Microbiological Evaluation of Diabetic Foot Syndrome. Medicina.

[10] Yang L., Rong G., Wu Q. (2022). Diabetic foot ulcer: Challenges and future. World J. Diabetes.

[11] Ramirez-Acuña J.M., Cardenas-Cadena S.A., Marquez-Salas P.A., Garza-Veloz I., Perez-Favila A., Cid-Baez M.A., Flores-Morales V., Martinez-Fierro M.L. (2019). Diabetic foot ulcers: Current advances in antimicrobial therapies and emerging treatments. Antibiotics.

[12] Apelqvist J. (2012). Diagnostics and treatment of the diabetic foot. Endocrine.

[13] Reardon R., Simring D., Kim B., Mortensen J., Williams D., Leslie A. (2020). The diabetic foot ulcer. Aust. J. Gen. Pract..

[14] Musuuza J., Sutherland B.L., Kurter S., Balasubramanian P., Bartels C.M., Brennan M.B. (2020). A systematic review of multidisciplinary teams to reduce major amputations for patients with diabetic foot ulcers. J. Vasc. Surg..

[15] Wang A., Lv G., Cheng X., Ma X., Wang W., Gui J., Hu J., Lu M., Chu G., Jin’an C. (2020). Guidelines on multidisciplinary approaches for the prevention and management of diabetic foot disease (2020 edition). Burn. Trauma.

[16] Perez-Favila A., Martinez-Fierro M.L., Rodriguez-Lazalde J.G., Cid-Baez M.A., Zamudio-Osuna M.D.J., Martinez-Blanco M.D.R., Mollinedo-Montaño F.E., Rodriguez-Sanchez I.P., Castañeda-Miranda R., Garza-Veloz I. (2019). Current therapeutic strategies in diabetic foot ulcers. Medicina.

[17] Sen P., Demirdal T., Emir B. (2019). Meta-analysis of risk factors for amputation in diabetic foot infections. Diabetes. Metab. Res. Rev..

[18] Dörr S., Freier F., Schlecht M., Lobmann R. (2021). Bacterial diversity and inflammatory response at first-time visit in younger and older individuals with diabetic foot infection (DFI). Acta Diabetol..

[19] Naomi R., Bahari H., Ridzuan P.M., Othman F. (2021). Natural-Based Biomaterial for Skin Wound Healing (Gelatin vs. Collagen): Expert Review. Polymers.

[20] Ng I.C., Pawijit P., Tan J., Yu H., Narayan R.B.T. (2019). Anatomy and Physiology for Biomaterials Research and Development. Encyclopedia of Biomedical Engineering.

[21] Divyashri G., Badhe R.V., Sadanandan B., Vijayalakshmi V., Kumari M., Ashrit P., Bijukumar D., Mathew M.T., Shetty K., Raghu A.V. (2022). Applications of hydrogel-based delivery systems in wound care and treatment: An up-to-date review. Polym. Adv. Technol..

[22] Güiza-Argüello V.R., Solarte-David V.A., Pinzón-Mora A.V., Ávila-Quiroga J.E., Becerra-Bayona S.M. (2022). Current Advances in the Development of Hydrogel-Based Wound Dressings for Diabetic Foot Ulcer Treatment. Polymers.

[23] Alven S., Peter S., Mbese Z., Aderibigbe B.A. (2022). Polymer-Based Wound Dressing Materials Loaded with Bioactive Agents: Potential Materials for the Treatment of Diabetic Wounds. Polymers.

[24] Bardill J.R., Laughter M.R., Stager M., Liechty K.W., Krebs M.D., Zgheib C. (2022). Topical gel-based biomaterials for the treatment of diabetic foot ulcers. Acta Biomater..

[25] Tallapaneni V., Kalaivani C., Pamu D., Mude L., Singh S.K., Karri V.V.S.R. (2021). Acellular Scaffolds as Innovative Biomaterial Platforms for the Management of Diabetic Wounds. Tissue Eng. Regen. Med..

[26] Srivastava P., Sondak T., Sivashanmugam K., Kim K. (2022). A Review of Immunomodulatory Reprogramming by Probiotics in Combating Chronic and Acute Diabetic Foot Ulcers (DFUs). Pharmaceutics.

[27] Chemello G., Salvatori B., Morettini M., Tura A. (2022). Artificial Intelligence Methodologies Applied to Technologies for Screening, Diagnosis and Care of the Diabetic Foot: A Narrative Review. Biosensors.

[28] Baig M.S., Banu A., Zehravi M., Rana R., Burle S.S., Khan S.L., Islam F., Siddiqui F.A., Massoud E.E.S., Rahman M.H. (2022). An Overview of Diabetic Foot Ulcers and Associated Problems with Special Emphasis on Treatments with Antimicrobials. Life.

[29] Saluja S., Anderson S.G., Hambleton I., Shoo H., Livingston M., Jude E.B., Lunt M., Dunn G., Heald A.H. (2020). Foot ulceration and its association with mortality in diabetes mellitus: A meta-analysis. Diabet. Med..

[30] Rubio J.A., Jiménez S., Lázaro-Martínez J.L. (2020). Mortality in patients with diabetic foot ulcers: Causes, risk factors, and their association with evolution and severity of ulcer. J. Clin. Med..

[31] Chammas N.K., Hill R.L.R., Edmonds M.E. (2016). Increased Mortality in Diabetic Foot Ulcer Patients: The Significance of Ulcer Type. J. Diabetes Res..

[32] Brownrigg J.R.W., Griffin M., Hughes C.O., Jones K.G., Patel N., Thompson M.M., Hinchliffe R.J. (2014). Influence of foot ulceration on cause-specific mortality in patients with diabetes mellitus. J. Vasc. Surg..

[33] Megallaa M.H., Ismail A.A., Zeitoun M.H., Khalifa M.S. (2019). Association of diabetic foot ulcers with chronic vascular diabetic complications in patients with type 2 diabetes. Diabetes Metab. Syndr. Clin. Res. Rev..

[34] Kalan L.R., Meisel J.S., Loesche M.A., Horwinski J., Soaita I., Chen X., Uberoi A., Gardner S.E., Grice E.A. (2019). Strain- and Species-Level Variation in the Microbiome of Diabetic Wounds Is Associated with Clinical Outcomes and Therapeutic Efficacy. Cell Host Microbe.

[35] Kareliya H., Bichile L., Bal A., Varaiya A., Bhalekar P. (2019). Fungal Infection in Diabetic Foot a Clinicomicrobiological Study. Acta Sci. Mcrobiol..

[36] Kalshetti V.T., Wadile R., Bothikar S.T., Ambade V., Bhate V.M. (2017). Study of fungal infections in diabetic foot Ulcer. Indian J. Microbiol. Res..

[37] Raiesi O., Shabandoust H., Dehghan P., Shamsaei S., Soleimani A. (2018). Fungal infection in foot diabetic patients. J. Basic Res. Med. Sci..

[38] Chellan G., Shivaprakash S., Ramaiyar S.K., Varma A.K., Varma N., Sukumaran M.T., Vasukutty J.R., Bal A., Kumar H. (2010). Spectrum and prevalence of fungi infecting deep tissues of lower-limb wounds in patients with type 2 diabetes. J. Clin. Microbiol..

[39] Ibrahim A., Berkache M., Morency-Potvin P., Juneau D., Koenig M., Bourduas K., Freire V. (2022). Diabetic foot infections: How to investigate more efficiently? A retrospective study in a quaternary university center. Insights Imaging.

[40] Rodrigues M., Kosaric N., Bonham C.A., Gurtner G.C. (2019). Wound healing: A cellular perspective. Physiol. Rev..

[41] Childs D.R., Murthy A.S. (2017). Overview of Wound Healing and Management. Surg. Clin. N. Am..

[42] Petkovic M., Sørensen A.E., Leal E.C., Carvalho E., Dalgaard L.T. (2020). Mechanistic Actions of microRNAs in Diabetic Wound Healing. Cells.

[43] Petkovic M., Vangmouritzen M., Mojsoska B., Jenssen H. (2021). Immunomodulatory properties of host defence peptides in skin wound healing. Biomolecules.

[44] Erem C., Hacıhasanoğlu A., Çelik Ş., Ovalı E., Ersöz H.Ö., Ukinç K., Deger O., Telatar M. (2005). Coagulation and Fibrinolysis Parameters in Type 2 Diabetic Patients with and without Diabetic Vascular Complications. Med. Princ. Pract..

[45] Xiao J., Li J., Cai L., Chakrabarti S., Li X. (2014). Cytokines and Diabetes Research. J. Diabetes Res..

[46] Santoro M.M., Gaudino G. (2005). Cellular and molecular facets of keratinocyte reepithelization during wound healing. Exp. Cell Res..

[47] Lan C.E., Liu I., Fang A., Wen C., Wu C. (2008). Hyperglycaemic conditions decrease cultured keratinocyte mobility: Implications for impaired wound healing in patients with diabetes. Br. J. Dermatol..

[48] Galiano R.D., Tepper O.M., Pelo C.R., Bhatt K.A., Callaghan M., Bastidas N., Bunting S., Steinmetz H.G., Gurtner G.C. (2004). Topical Vascular Endothelial Growth Factor Accelerates Diabetic Wound Healing through Increased Angiogenesis and by Mobilizing and Recruiting Bone Marrow-Derived Cells. Am. J. Pathol..

[49] Maione A.G., Smith A., Kashpur O., Yanez V., Knight E., Mooney D.J., Veves A., Tomic-Canic M., Garlick J.A. (2016). Altered ECM deposition by diabetic foot ulcer-derived fibroblasts implicates fibronectin in chronic wound repair. Wound Repair Regen..

[50] Lazzarini P., Fernando M., Van Netten J. (2019). Diabetic foot ulcers: Is remission a realistic goal?. Endocrinol. Today.

[51] Da Porto A., Miranda C., Brosolo G., Zanette G., Michelli A., Ros R. (2022). Da Nutritional supplementation on wound healing in diabetic foot: What is known and what is new?. World J. Diabetes.

[52] Chuan F., Tang K., Jiang P., Zhou B., He X. (2015). Reliability and Validity of the Perfusion, Extent, Depth, Infection and Sensation (PEDIS) Classification System and Score in Patients with Diabetic Foot Ulcer. PLoS ONE.

[53] Biz C., Ruggieri P. (2023). Minimally Invasive Surgery: Osteotomies for Diabetic Foot Disease. Foot Ankle Clin..

[54] Biz C., Belluzzi E., Crimì A., Bragazzi N.L., Nicoletti P., Mori F., Ruggieri P. (2021). Minimally Invasive Metatarsal Osteotomies (MIMOs) for the Treatment of Plantar Diabetic Forefoot Ulcers (PDFUs): A Systematic Review and Meta-Analysis with Meta-Regressions. Appl. Sci..

[55] Pombeiro I., Moura J., Pereira M.G., Carvalho E. (2022). Stress-Reducing Psychological Interventions as Adjuvant Therapies for Diabetic Chronic Wounds. Curr. Diabetes Rev..

[56] Pereira M.G., Vilaça M., Carvalho E. (2022). Effectiveness of Two Stress Reduction Interventions in Patients with Chronic Diabetic Foot Ulcers (PSY-DFU): Protocol for a Longitudinal RCT with a Nested Qualitative Study Involving Family Caregivers. Int. J. Environ. Res. Public Health.

[57] Armstrong D.G., Boulton A.J.M., Bus S.A. (2017). Diabetic Foot Ulcers and Their Recurrence. N. Engl. J. Med..

[58] Fu X.-L., Ding H., Miao W.-W., Mao C.-X., Zhan M.-Q., Chen H.-L. (2019). Global recurrence rates in diabetic foot ulcers: A systematic review and meta-analysis. Diabetes. Metab. Res. Rev..

[59] Alven S., Aderibigbe B.A. (2020). Chitosan and Cellulose-Based Hydrogels for Wound Management. Int. J. Mol. Sci..

[60] Su J., Li J., Liang J., Zhang K., Li J. (2021). Hydrogel Preparation Methods and Biomaterials for Wound Dressing. Life.

[61] Blanco-Fernandez B., Castaño O., Mateos-Timoneda M.Á., Engel E., Pérez-Amodio S. (2021). Nanotechnology Approaches in Chronic Wound Healing. Adv. Wound Care.

[62] Chen H., Cheng Y., Tian J., Yang P., Zhang X., Chen Y., Hu Y., Wu J. (2020). Dissolved oxygen from microalgae-gel patch promotes chronic wound healing in diabetes. Sci. Adv..

[63] Li S., Wang X., Chen J., Guo J., Yuan M., Wan G., Yan C., Li W., Machens H.-G., Rinkevich Y. (2022). Calcium ion cross-linked sodium alginate hydrogels containing deferoxamine and copper nanoparticles for diabetic wound healing. Int. J. Biol. Macromol..

[64] Ji S., Zhou S., Zhang X., Chen W., Jiang X. (2022). An oxygen-sensitive probe and a hydrogel for optical imaging and photodynamic antimicrobial chemotherapy of chronic wounds. Biomater. Sci..

[65] Azarniya A., Tamjid E., Eslahi N., Simchi A. (2019). Modification of bacterial cellulose/keratin nanofibrous mats by a tragacanth gum-conjugated hydrogel for wound healing. Int. J. Biol. Macromol..

[66] Pessanha F.S., Oliveira B.G., Oliveira B.C., Deutsch G., Teixeira F.L., Bokehi L.C., Calomino M.A., Rodrigues de Castilho S., Thiré R.M., Teixeira L.A. (2023). Effectiveness of Epidermal Growth Factor Loaded Carboxymethylcellulose (EGF-CMC) Hydrogel in Biofilm Formation in Wounds of Diabetic Patients: A Randomized Clinical Trial. Gels.

[67] Pan W., Qi X., Xiang Y., You S., Cai E., Gao T., Tong X., Hu R., Shen J., Deng H. (2022). Facile formation of injectable quaternized chitosan/tannic acid hydrogels with antibacterial and ROS scavenging capabilities for diabetic wound healing. Int. J. Biol. Macromol..

[68] Zhou A., Zhang Y., Zhang X., Deng Y., Huang D., Huang C., Qu Q. (2022). Quaternized chitin/tannic acid bilayers layer-by-layer deposited poly(lactic acid)/polyurethane nanofibrous mats decorated with photoresponsive complex and silver nanoparticles for antibacterial activity. Int. J. Biol. Macromol..

[69] Lee Y.-H., Lin S.-J. (2022). Chitosan/PVA Hetero-Composite Hydrogel Containing Antimicrobials, Perfluorocarbon Nanoemulsions, and Growth Factor-Loaded Nanoparticles as a Multifunctional Dressing for Diabetic Wound Healing: Synthesis, Characterization, and In Vitro/In Vivo Evaluation. Pharmaceutics.

[70] Ma S., Hu H., Wu J., Li X., Ma X., Zhao Z., Liu Z., Wu C., Zhao B., Wang Y. (2022). Functional extracellular matrix hydrogel modified with MSC-derived small extracellular vesicles for chronic wound healing. Cell Prolif..

[71] Jirofti N., Golandi M., Movaffagh J., Ahmadi F.S., Kalalinia F. (2021). Improvement of the Wound-Healing Process by Curcumin-Loaded Chitosan/Collagen Blend Electrospun Nanofibers: In Vitro and In Vivo Studies. ACS Biomater. Sci. Eng..

[72] Wei S., Xu P., Yao Z., Cui X., Lei X., Li L., Dong Y., Zhu W., Guo R., Cheng B. (2021). A composite hydrogel with co-delivery of antimicrobial peptides and platelet-rich plasma to enhance healing of infected wounds in diabetes. Acta Biomater..

[73] Guo C., Wu Y., Li W., Wang Y., Kong Q. (2022). Development of a Microenvironment-Responsive Hydrogel Promoting Chronically Infected Diabetic Wound Healing through Sequential Hemostatic, Antibacterial, and Angiogenic Activities. ACS Appl. Mater. Interfaces.

[74] Wu S., Yang Y., Wang S., Dong C., Zhang X., Zhang R., Yang L. (2022). Dextran and peptide-based pH-sensitive hydrogel boosts healing process in multidrug-resistant bacteria-infected wounds. Carbohydr. Polym..

[75] Nilforoushzadeh M.A., Sisakht M.M., Amirkhani M.A., Seifalian A.M., Banafshe H.R., Verdi J., Nouradini M. (2020). Engineered skin graft with stromal vascular fraction cells encapsulated in fibrin–collagen hydrogel: A clinical study for diabetic wound healing. J. Tissue Eng. Regen. Med..

[76] Zhu H., Luo H., Lin M., Li Y., Chen A., He H., Sheng F., Wu J. (2022). Methacrylated gelatin shape-memorable cryogel subcutaneously delivers EPCs and aFGF for improved pressure ulcer repair in diabetic rat model. Int. J. Biol. Macromol..

[77] Shi M., Gao Y., Lee L., Song T., Zhou J., Yan L., Li Y. (2022). Adaptive Gelatin Microspheres Enhanced Stem Cell Delivery and Integration With Diabetic Wounds to Activate Skin Tissue Regeneration. Front. Bioeng. Biotechnol..

[78] Xiong Y., Chen L., Liu P., Yu T., Lin C., Yan C., Hu Y., Zhou W., Sun Y., Panayi A.C. (2022). All-in-One: Multifunctional Hydrogel Accelerates Oxidative Diabetic Wound Healing through Timed-Release of Exosome and Fibroblast Growth Factor. Small.

[79] Xu Z., Liu G., Huang J., Wu J. (2022). Novel Glucose-Responsive Antioxidant Hybrid Hydrogel for Enhanced Diabetic Wound Repair. ACS Appl. Mater. Interfaces.

[80] Alihosseini F., Sun G. (2016). Plant-based compounds for antimicrobial textiles. Antimicrobial Textiles.

[81] Chia J.J., Shameli K., Yusefi M., Ali R.R., Balasundram V., Teow S.-Y. (2022). Preparation and Application of Cross-linked Alginate Nanoparticles as Drug Carrier: A Review. J. Res. Nanosci. Nanotechnol..

[82] Stoica A.E., Chircov C., Grumezescu A.M. (2020). Nanomaterials for Wound Dressings: An Up-to-Date Overview. Molecules.

[83] Rosiak P., Latanska I., Paul P., Sujka W., Kolesinska B. (2021). Modification of Alginates to Modulate Their Physic-Chemical Properties and Obtain Biomaterials with Different Functional Properties. Molecules.

[84] Barbu A., Neamtu B., Zăhan M., Iancu G.M., Bacila C., Mireșan V. (2021). Current Trends in Advanced Alginate-Based Wound Dressings for Chronic Wounds. J. Pers. Med..

[85] Zhang M., Zhao X. (2020). Alginate hydrogel dressings for advanced wound management. Int. J. Biol. Macromol..

[86] Qin Y. (2004). Gel swelling properties of alginate fibers. J. Appl. Polym. Sci..

[87] Teixeira M.O., Antunes J.C., Felgueiras H.P. (2021). Recent Advances in Fiber-Hydrogel Composites for Wound Healing and Drug Delivery Systems. Antibiotics.

[88] Straccia M.C., D’Ayala G.G., Romano I., Oliva A., Laurienzo P. (2015). Alginate Hydrogels Coated with Chitosan for Wound Dressing. Mar. Drugs.

[89] Roe D.F., Gibbins B.L., Ladizinsky D.A. (2010). Topical Dissolved Oxygen Penetrates Skin: Model and Method. J. Surg. Res..

[90] Singla R., Soni S., Patial V., Kulurkar P.M., Kumari A., Mahesh S., Padwad Y.S., Yadav S.K. (2017). In vivo diabetic wound healing potential of nanobiocomposites containing bamboo cellulose nanocrystals impregnated with silver nanoparticles. Int. J. Biol. Macromol..

[91] Singla R., Soni S., Patial V., Kulurkar P., Kumari A., Mahesh S., Padwad Y., Yadav S. (2017). Cytocompatible Anti-microbial Dressings of Syzygium cumini Cellulose Nanocrystals Decorated with Silver Nanoparticles Accelerate Acute and Diabetic Wound Healing. Sci. Rep..

[92] Grip J., Engstad R.E., Skjæveland I., Škalko-Basnet N., Isaksson J., Basnet P., Holsæter A.M. (2018). Beta-glucan-loaded nanofiber dressing improves wound healing in diabetic mice. Eur. J. Pharm. Sci..

[93] Aranaz I., Alcántara A.R., Civera M.C., Arias C., Elorza B., Heras Caballero A., Acosta N. (2021). Chitosan: An Overview of Its Properties and Applications. Polymers.

[94] Matica M.A., Aachmann F.L., Tøndervik A., Sletta H., Ostafe V. (2019). Chitosan as a Wound Dressing Starting Material: Antimicrobial Properties and Mode of Action. Int. J. Mol. Sci..

[95] Singh R., Shitiz K., Singh A. (2017). Chitin and chitosan: Biopolymers for wound management. Int. Wound J..

[96] Hou S., Liu Y., Feng F., Zhou J., Feng X., Fan Y. (2020). Polysaccharide-Peptide Cryogels for Multidrug-Resistant-Bacteria Infected Wound Healing and Hemostasis. Adv. Healthc. Mater..

[97] Baidamshina D.R., Koroleva V.A., Trizna E.Y., Pankova S.M., Agafonova M.N., Chirkova M.N., Vasileva O.S., Akhmetov N., Shubina V.V., Porfiryev A.G. (2020). Anti-biofilm and wound-healing activity of chitosan-immobilized Ficin. Int. J. Biol. Macromol..

[98] Rodríguez-Acosta H., Tapia- Rivera J.M., Guerrero-Guzmán A., Hernández-Elizarraráz E., Hernández- Díaz J.A., Garza- García J.J.O., Pérez- Ramírez P.E., Velasco- Ramírez S.F., Ramírez- Anguiano A.C., Velázquez- Juárez G. (2022). Chronic wound healing by controlled release of chitosan hydrogels loaded with silver nanoparticles and calendula extract. J. Tissue Viability.

[99] Abueva C., Ryu H.S., Min J.W., Chung P.S., You H.S., Yang M.S., Woo S.H. (2021). Quaternary ammonium N,N,N-trimethyl chitosan derivative and povidone-iodine complex as a potent antiseptic with enhanced wound healing property. Int. J. Biol. Macromol..

[100] Wu J.-Y., Ooi C.W., Song C.P., Wang C.-Y., Liu B.-L., Lin G.-Y., Chiu C.-Y., Chang Y.-K. (2021). Antibacterial efficacy of quaternized chitosan/poly (vinyl alcohol) nanofiber membrane crosslinked with blocked diisocyanate. Carbohydr. Polym..

[101] Pan Z., Ye H., Wu D. (2021). Recent advances on polymeric hydrogels as wound dressings. APL Bioeng..

[102] Zhang Y., Wang Y., Li Y., Yang Y., Jin M., Lin X., Zhuang Z., Guo K., Zhang T., Tan W. (2023). Application of Collagen-Based Hydrogel in Skin Wound Healing. Gels.

[103] Mousavi S., Khoshfetrat A.B., Khatami N., Ahmadian M., Rahbarghazi R. (2019). Comparative study of collagen and gelatin in chitosan-based hydrogels for effective wound dressing: Physical properties and fibroblastic cell behavior. Biochem. Biophys. Res. Commun..

[104] Zarrintaj P., Saeb M.R., Jafari S.H., Mozafari M., Ajitha A.R., Thomas S. (2020). Chapter 18—Application of compatibilized polymer blends in biomedical fields. Compatibilization of Polymer Blends.

[105] Annu, Ahmed S., Ahmed S.B.T.-A.G.M. (2021). Advanced green materials: An overview. Woodhead Publishing in Materials.

[106] Lin S.-P., Lo K.-Y., Tseng T.-N., Liu J.-M., Shih T.-Y., Cheng K.-C. (2019). Evaluation of PVA/dextran/chitosan hydrogel for wound dressing. Cell. Polym..

[107] Murphy K.C., Whitehead J., Zhou D., Ho S.S., Leach J.K. (2017). Engineering fibrin hydrogels to promote the wound healing potential of mesenchymal stem cell spheroids. Acta Biomater..

[108] Schneider-Barthold C., Baganz S., Wilhelmi M., Scheper T., Pepelanova I. (2016). Hydrogels based on collagen and fibrin—Frontiers and applications. BioNanoMaterials.

[109] Coradin T., Wang K., Law T., Trichet L. (2020). Type I Collagen-Fibrin Mixed Hydrogels: Preparation, Properties and Biomedical Applications. Gels.

[110] Qi L., Zhang C., Wang B., Yin J., Yan S. (2022). Progress in Hydrogels for Skin Wound Repair. Macromol. Biosci..

[111] Wang Y., Wu Y., Long L., Yang L., Fu D., Hu C., Kong Q., Wang Y. (2021). Inflammation-Responsive Drug-Loaded Hydrogels with Sequential Hemostasis, Antibacterial, and Anti-Inflammatory Behavior for Chronically Infected Diabetic Wound Treatment. ACS Appl. Mater. Interfaces.

[112] Mogoşanu G.D., Grumezescu A.M. (2014). Natural and synthetic polymers for wounds and burns dressing. Int. J. Pharm..

[113] Jiang Y., Han Y., Wang J., Lv F., Yi Z., Ke Q., Xu H. (2019). Space-Oriented Nanofibrous Scaffold with Silicon-Doped Amorphous Calcium Phosphate Nanocoating for Diabetic Wound Healing. ACS Appl. Bio Mater..

[114] Cam M.E., Ertas B., Alenezi H., Hazar-Yavuz A.N., Cesur S., Ozcan G.S., Ekentok C., Guler E., Katsakouli C., Demirbas Z. (2021). Accelerated diabetic wound healing by topical application of combination oral antidiabetic agents-loaded nanofibrous scaffolds: An in vitro and in vivo evaluation study. Mater. Sci. Eng. C.

[115] Masood N., Ahmed R., Tariq M., Ahmed Z., Masoud M.S., Ali I., Asghar R., Andleeb A., Hasan A. (2019). Silver nanoparticle impregnated chitosan-PEG hydrogel enhances wound healing in diabetes induced rabbits. Int. J. Pharm..

[116] Wu C., Long L., Zhang Y., Xu Y., Lu Y., Yang Z., Guo Y., Zhang J., Hu X., Wang Y. (2022). Injectable conductive and angiogenic hydrogels for chronic diabetic wound treatment. J. Control. Release.

[117] Xu Z., Liu Y., Ma R., Chen J., Qiu J., Du S., Li C., Wu Z., Yang X., Chen Z. (2022). Thermosensitive Hydrogel Incorporating Prussian Blue Nanoparticles Promotes Diabetic Wound Healing via ROS Scavenging and Mitochondrial Function Restoration. ACS Appl. Mater. Interfaces.

[118] Yu H., Li Y., Pan Y., Wang H., Wang W., Ren X., Yuan H., Lv Z., Zuo Y., Liu Z. (2023). Multifunctional porous poly (L-lactic acid) nanofiber membranes with enhanced anti-inflammation, angiogenesis and antibacterial properties for diabetic wound healing. J. Nanobiotechnol..

[119] Ali I.H., Khalil I.A., El-Sherbiny I.M. (2023). Design, development, in-vitro and in-vivo evaluation of polylactic acid-based multifunctional nanofibrous patches for efficient healing of diabetic wounds. Sci. Rep..

[120] Di Cristo F., Valentino A., De Luca I., Peluso G., Bonadies I., Di Salle A., Calarco A. (2023). Polylactic Acid/Poly(vinylpyrrolidone) Co-Electrospun Fibrous Membrane as a Tunable Quercetin Delivery Platform for Diabetic Wounds. Pharmaceutics.

[121] Zheng Z., Liu Y., Huang W., Mo Y., Lan Y., Guo R., Cheng B. (2018). Neurotensin-loaded PLGA/CNC composite nanofiber membranes accelerate diabetic wound healing. Artif. Cells Nanomed. Biotechnol..

[122] Hasan N., Cao J., Lee J., Naeem M., Hlaing S.P., Kim J., Jung Y., Lee B.-L., Yoo J.-W. (2019). PEI/NONOates-doped PLGA nanoparticles for eradicating methicillin-resistant *Staphylococcus aureus* biofilm in diabetic wounds via binding to the biofilm matrix. Mater. Sci. Eng. C.

[123] Lv H., Zhao M., Li Y., Li K., Chen S., Zhao W., Wu S., Han Y. (2022). Electrospun Chitosan&ndash;Polyvinyl Alcohol Nanofiber Dressings Loaded with Bioactive Ursolic Acid Promoting Diabetic Wound Healing. Nanomaterials.

[124] Ningrum D.R., Hanif W., Mardhian D.F., Asri L.A.T.W. (2023). In Vitro Biocompatibility of Hydrogel Polyvinyl Alcohol/Moringa oleifera Leaf Extract/Graphene Oxide for Wound Dressing. Polymers.

[125] Contardi M., Summa M., Picone P., Brancato O.R., Di Carlo M., Bertorelli R., Athanassiou A. (2022). Evaluation of a Multifunctional Polyvinylpyrrolidone/Hyaluronic Acid-Based Bilayer Film Patch with Anti-Inflammatory Properties as an Enhancer of the Wound Healing Process. Pharmaceutics.

[126] Yu J., Wang P., Yin M., Zhang K., Wang X., Han B. (2022). Carboxymethyl chitosan-grafted polyvinylpyrrolidone-iodine microspheres for promoting the healing of chronic wounds. Bioengineered.

[127] Ranjbar-Mohammadi M., Rabbani S., Bahrami S.H., Joghataei M.T., Moayer F. (2016). Antibacterial performance and in vivo diabetic wound healing of curcumin loaded gum tragacanth/poly(ε-caprolactone) electrospun nanofibers. Mater. Sci. Eng. C.

[128] Lv F., Wang J., Xu P., Han Y., Ma H., Xu H., Chen S., Chang J., Ke Q., Liu M. (2017). A conducive bioceramic/polymer composite biomaterial for diabetic wound healing. Acta Biomater..

[129] Zhong X., Ji C., Chan A.K.L., Kazarian S.G., Ruys A., Dehghani F. (2011). Fabrication of chitosan/poly(ε-caprolactone) composite hydrogels for tissue engineering applications. J. Mater. Sci. Mater. Med..

[130] Yang G., Lin H., Rothrauff B.B., Yu S., Tuan R.S. (2016). Multilayered polycaprolactone/gelatin fiber-hydrogel composite for tendon tissue engineering. Acta Biomater..

[131] Lara H.H., Garza-Treviño E.N., Ixtepan-Turrent L., Singh D.K. (2011). Silver nanoparticles are broad-spectrum bactericidal and virucidal compounds. J. Nanobiotechnology.

[132] Yamaguchi Y., Li Z., Zhu X., Liu C., Zhang D., Dou X. (2015). Correction: Polyethylene Oxide (PEO) and Polyethylene Glycol (PEG) Polymer Sieving Matrix for RNA Capillary Electrophoresis. PLoS ONE.

[133] Gelardi G., Mantellato S., Marchon D., Palacios M., Eberhardt A.B., Flatt R.J., Aïtcin P.-C., Flatt R.J.B.T. (2016). Chemistry of chemical admixtures. Science and Technology of Concrete Admixtures.

[134] Theodosopoulos G.V., Zisis C., Charalambidis G., Nikolaou V., Coutsolelos A.G., Pitsikalis M. (2017). Synthesis, Characterization and Thermal Properties of Poly(ethylene oxide), PEO, Polymacromonomers via Anionic and Ring Opening Metathesis Polymerization. Polymers.

[135] Wong R.S.H., Dodou K. (2017). Effect of Drug Loading Method and Drug Physicochemical Properties on the Material and Drug Release Properties of Poly (Ethylene Oxide) Hydrogels for Transdermal Delivery. Polymers.

[136] Karkri M., Sadasivuni K.K., Ponnamma D., Kim J., Cabibihan J.-J., AlMaadeed M.A.B.T. (2017). Thermal Conductivity of Biocomposite Materials. Biopolymer Composites in Electronics.

[137] Nath P.C., Nandi N.B., Tiwari A., Das J., Roy B., Sharma A., Vijayakumar P.S., Prabhakar E.P.K., Kumar R.B.T. (2023). Chapter 17—Applications of nanotechnology in food sensing and food packaging. Nanotechnology Applications for Food Safety and Quality Monitoring.

[138] Little A., Wemyss A.M., Haddleton D.M., Tan B., Sun Z., Ji Y., Wan C. (2021). Synthesis of Poly(Lactic Acid-co-Glycolic Acid) Copolymers with High Glycolide Ratio by Ring-Opening Polymerisation. Polymers.

[139] Mo Y., Guo R., Liu J., Lan Y., Zhang Y., Xue W., Zhang Y. (2015). Preparation and properties of PLGA nanofiber membranes reinforced with cellulose nanocrystals. Colloids Surf. B Biointerfaces.

[140] Kurakula M., Rao G.S.N.K. (2020). Pharmaceutical assessment of polyvinylpyrrolidone (PVP): As excipient from conventional to controlled delivery systems with a spotlight on COVID-19 inhibition. J. Drug Deliv. Sci. Technol..

[141] Kurakula M., Koteswara Rao G.S.N. (2020). Moving polyvinyl pyrrolidone electrospun nanofibers and bioprinted scaffolds toward multidisciplinary biomedical applications. Eur. Polym. J..

[142] Contardi M., Russo D., Suarato G., Heredia-Guerrero J.A., Ceseracciu L., Penna I., Margaroli N., Summa M., Spanò R., Tassistro G. (2019). Polyvinylpyrrolidone/hyaluronic acid-based bilayer constructs for sequential delivery of cutaneous antiseptic and antibiotic. Chem. Eng. J..

[143] Laurano R., Boffito M., Ciardelli G., Chiono V. (2022). Wound dressing products: A translational investigation from the bench to the market. Eng. Regen..

[144] Barakat M., DiPietro L.A., Chen L. (2020). Limited Treatment Options for Diabetic Wounds: Barriers to Clinical Translation Despite Therapeutic Success in Murine Models. Adv. Wound Care.

[145] Rizzi S.C., Upton Z., Bott K., Dargaville T.R. (2010). Recent advances in dermal wound healing: Biomedical device approaches. Expert Rev. Med. Devices.

[146] Milne J. (2016). The challenge of providing cost-effective wound care. Wounds.

[147] Rossing P. (2020). Bone Marrow Derived Allogeneic Mesenchymal Stromal Cells to Non-healing Diabetic Foot Wounds (REDDSTAR).

[148] Yarahmadi A., Saeed Modaghegh M.-H., Mostafavi-Pour Z., Azarpira N., Mousavian A., Bonakdaran S., Jarahi L., Samadi A., Peimani M., Hamidi Alamdari D. (2021). The effect of platelet-rich plasma-fibrin glue dressing in combination with oral vitamin E and C for treatment of non-healing diabetic foot ulcers: A randomized, double-blind, parallel-group, clinical trial. Expert Opin. Biol. Ther..

[149] Hamidi Alamdari D. (2020). The Effect of Platelet-Rich Plasma-Fibrin Glue in Combination with Vitamin E and C for Treatment of Non-Healing Diabetic Foot Ulcers.

[150] Grek C.L., Prasad G.M., Viswanathan V., Armstrong D.G., Gourdie R.G., Ghatnekar G.S. (2015). Topical administration of a connexin43-based peptide augments healing of chronic neuropathic diabetic foot ulcers: A multicenter, randomized trial. Wound Repair Regen..

[151] Xequel Bio, Inc. (2020). A Study of Granexin Gel in the Treatment of Diabetic Foot Ulcer.

[152] Mrozikiewicz-Rakowska B., Szabłowska-Gadomska I., Cysewski D., Rudziński S., Płoski R., Gasperowicz P., Konarzewska M., Zieliński J., Mieczkowski M., Sieńko D. (2023). Allogenic Adipose-Derived Stem Cells in Diabetic Foot Ulcer Treatment: Clinical Effectiveness, Safety, Survival in the Wound Site, and Proteomic Impact. Int. J. Mol. Sci..

[153] Mrozikiewicz-Rakowska B. (2021). Treatment of Chronic Wounds in Diabetic Foot Syndrome with Allogeneic Adipose Derived Mesenchymal Stem Cells (1ABC).

[154] Keeley D. (2021). A Prospective, Randomized Clinical Trial of PRP Concepts Fibrin Bio-Matrix in Non-Healing Diabetic Foot Ulcers.

[155] Khan Z. (2022). Evaluating the Effectiveness of Piscean Derived Collagen Dressing on Neuropathic Diabetic Foot Ulcer in T2DM Patients.

[156] Omeza, LLC (2022). Omeza Products in Combination with Standard of Care for the Treatment of Diabetic Foot Ulcers.

[157] Serena T., Moore S. (2022). Clinical Trial Evaluating a Hyaluronic Acid Matrix in the Treatment of Chronic Non-Healing Diabetic Foot Ulcers (DFUs).

[158] Serena T., Witkowski J. (2022). Effect of Meso Wound Matrix in the Treatment of DFUs.

[159] Al-Jalodi O., Sabo M., Patel K., Bullock N., Serena L., Breisinger K., Serena T.E. (2021). Efficacy and safety of a porcine peritoneum-derived matrix in diabetic foot ulcer treatment: A pilot study. J. Wound Care.

[160] Ho C. (2023). Pivotal Study of an Antimicrobial Wound Dressing to Treat Chronic Wounds.

[161] Rihter Z. (2023). An Open, Non-Comparative, Multicenter Investigation to Evaluate the Safety and Performance of Exufiber Ag^+^, a Gelling Fiber Silver Dressing, When Used in Medium to High Exuding Chronic Wounds.

[162] Oropallo A. (2021). Pilot Study of PHOENIX Impact on Wound Microbiome.

[163] Rivellese A. (2022). Fitostimoline^®^ Hydrogel Versus Saline Gauze Dressing in Diabetic Foot Ulcers.

[164] Vigfusdottir S. (2022). ChitoCare Medical Wound Healing Gel PMCF Study on Healing of Chronic Wounds (CHITOCHRONIC).

[165] Böhling A. (2023). Study to Examine Clinical Performance and Safety of Cutimed^®^ Gelling Fiber in Routine Clinical Practice (GELFI).

